# Linked shrinkage to improve estimation of interaction effects in regression models

**DOI:** 10.1515/em-2023-0039

**Published:** 2024-07-09

**Authors:** Mark A. van de Wiel, Matteo Amestoy, Jeroen Hoogland

**Affiliations:** Department of Epidemiology and Data Science, Amsterdam Public Health Research Institute, Amsterdam University Medical Centers, Amsterdam, The Netherlands

**Keywords:** regression, interactions, shrinkage, variable importance, Shapley values

## Abstract

**Objectives:**

The addition of two-way interactions is a classic problem in statistics, and comes with the challenge of quadratically increasing dimension. We aim to a) devise an estimation method that can handle this challenge and b) to aid interpretation of the resulting model by developing computational tools for quantifying variable importance.

**Methods:**

Existing strategies typically overcome the dimensionality problem by only allowing interactions between relevant main effects. Building on this philosophy, and aiming for settings with moderate n to p ratio, we develop a local shrinkage model that links the shrinkage of interaction effects to the shrinkage of their corresponding main effects. In addition, we derive a new analytical formula for the Shapley value, which allows rapid assessment of individual-specific variable importance scores and their uncertainties.

**Results:**

We empirically demonstrate that our approach provides accurate estimates of the model parameters and very competitive predictive accuracy. In our Bayesian framework, estimation inherently comes with inference, which facilitates variable selection. Comparisons with key competitors are provided. Large-scale cohort data are used to provide realistic illustrations and evaluations. The implementation of our method in RStan is relatively straightforward and flexible, allowing for adaptation to specific needs.

**Conclusions:**

Our method is an attractive alternative for existing strategies to handle interactions in epidemiological and/or clinical studies, as its linked local shrinkage can improve parameter accuracy, prediction and variable selection. Moreover, it provides appropriate inference and interpretation, and may compete well with less interpretable machine learners in terms of prediction.

## Introduction

Adding interactions to a regression model is a classical problem in epidemiology which may lead to interesting insights on the joint effects of covariates [1]. Moreover, it may be crucial for reducing bias of the main effect coefficients [2]. It comes at a price though, as the number of interaction terms *q* increases quadratically with the number of covariates *p*. While one may argue that in very small *p* settings the plausibility of each two-way interaction may be considered separately, such a strategy is infeasible or unpractical for larger dimensions. At the other end of the spectrum, with *p* large – and hence *q* very large – compared to sample size *n*, the hierarchical lasso [3] and variations thereof provide a computationally efficient sparse solution. The latter, however, focuses on selection, and does not come naturally with parameter inference. This leaves a gap for the middle spectrum, with *p* + *q* of a similar order of magnitude as *n*, a setting which is fairly common in many biomedical or epidemiological studies. Our goal is to fill this gap using an interpretable solution that on one hand is able to deal with the large number of parameters, while on the other hand allows for inference. More specifically, our aim is three-fold: (1) accurate estimation and selection of parameters; (2) interpretation of variable importance scores in the context of our model; and (3) inference for those variable importance scores.

To achieve these goals, this study provides two novelties. First, a linked shrinkage model, which links local shrinkage of the interaction effects to that of the main effects. This extends the Bayesian local shrinkage framework [4]. The latter provides flexible, differential shrinkage of small and large effects, which may benefit the accuracy of the parameter estimation in the same spirit as the adaptive lasso and non-negative garrotte do. In addition, we draw upon its good inferential properties [5]. The linked shrinkage model also includes a global shrinkage parameter for the interaction parameters to allow those to be weaker on average than the main effect parameters, thereby providing adaptivity. Second, we deduce a computationally efficient equation for Shapley values [6], which allows quantification and inference for those sample specific variable importance scores. Shapley values are popular in machine learning, and we argue that these scores can also be of great use for regression models with many interaction terms, as the presence of the latter impedes straightforward interpretation of the regression coefficients as variable importance scores [1].

As our problem is a classical one in epidemiology, a number of solutions already exist. Below we provide a list of reference methods that we compare our method to.

– Ordinary least squares (OLS), which does not apply any shrinkage, and may therefore provide unstable estimates for some settings.
– Ridge regression with two tuned penalties [7], one for main effects, one for interactions: ridge2. Such global penalties likely improve the predictive abilities of the model, but do not well accommodate strong differences between parameter strengths within each of the two parameter sets.
– Bayesian local shrinkage [4] using a local Gaussian prior for each parameter, the standard deviations of which are endowed with a half-Cauchy prior. For our comparisons, we use the horseshoe (hs; [8]), which extends the local shrinkage by a global shrinkage parameter to better accommodate sparse settings.
– Two-step approach that only include interactions of significant main effects: 2step. While popular in practice, it may render very unstable results, as the inclusion of interactions depends on a hard threshold for the main effects.
– Spike-and-slab (SandS; [9]), a prior that specificially models selection as it consists of a point mass on zero and, in our case, a Gaussian slab to model the non-zero effects.
– Lasso regression with only a penalty for the interaction terms: lassoint. This type of global shrinkage may suffer from the same drawbacks as ridge2.
– Adaptive lasso (adlasso; [10]), which weighs the penalties by reciprocal absolute OLS coefficients. Hence, its performance depends also on the stability of those coefficients.
– Hierarchical lasso for interactions (hlasso), a state-of-the-art methodology that formalizes the reasoning of 2step in one fitting procedure [3, 11, 12]. It is mostly designed for computational efficiency to handle large *p*. While it has proven its use for variable selection, formal inference is far from straightforward [11], requiring strong assumptions on the underlying sparsity or extensive resampling.

We compare our linked shrinkage model, termed Bayint, to those methods as well as to several variations of Bayint which differ in how they encode the linked shrinkage. We assess estimation accuracy, prediction and variable selection. For this, we use simulations and a very large real data set, the OLS estimates of which serve as a benchmark. We study the results for two outcomes (systolic blood pressure and cholesterol), and a mix of continuous, binary and categorical covariates. In addition, we provide several illustrations to support interpretation of the model and the covariates, including those with Shapley values and their uncertainties. Finally, we perform a short comparison with random forest in terms of out-of-bag predictive performance. This illustrates that even for large sample sizes Bayint can be very competitive to such a machine learner, while providing better interpretation. We end by discussing the implementation, scalability and potential extensions.

## Materials and methods

The model, called Bayint, combines ideas behind the hierarchical lasso, which considers interactions of strong main effects to be more important, with those of Bayesian local shrinkage, the hierarchical set-up of which allows a softer link between the interactions and main effects.

### The linked shrinkage model

For simplicity, we assume linear response *Y*_*i*_, *i*=1, …, *n*, but the model can easily be reformulated in a generalized linear model or Cox regression context. For sample *i*, the *j*th covariate is denoted by *x*_*ij*_, *j*=1, …, *p*. Then, the proposed model is:

(1) $$\begin{array}{cc} Y_{i} & =\alpha +\overset{p}{\underset{j=1}{\sum }}\beta_{j}x_{ij}+\underset{j,k:j\neq k}{\sum }\beta_{jk}x_{ij}x_{ik}+\epsilon_{i}\\ \alpha & ∼N\left(0,10^{2}\right)\\ \beta_{j} & ∼N\left(0,\sigma^{2}\tau_{j}^{2}\right)\\ \beta_{jk} & ∼N\left(0,\sigma^{2}\tau_{j}\tau_{k}\tau_{\text{int}}\right)\\ \epsilon_{i} & ∼N\left(0,\sigma^{2}\right)\\ \tau_{j} & ∼C^{+}\left(0,1\right)\\ \tau_{\text{int}} & ∼U\left(0.01,1\right)\\ \sigma^{2} & ∼IG\left(1,0.001\right) \end{array}$$

Several of the components in (1) are in line with conventional Bayesian modelling, including the half-Cauchy prior on the (relative) standard deviations *τ*_*j*_ [4]. We add linked shrinkage to the model by including the product *τ*_*j*_ *τ*_*k*_ in the prior of *β*_*jk*_. This product renders a symmetric handling of strong and weak main effects (corresponding to large *τ*_*j*_ and small *τ*_*j*_, respectively), whereas it is on the same scale as each of the components when they are. In addition, *τ*_int_, 0.01≤*τ*_int_≤1 is a shrinkage parameter shared by all interactions that models the prior believe that interaction parameters might, on average, be weaker than the main effect parameters. The lower bound avoids complete shrinkage to 0 of all interaction effects, as this may be undesirable in a sparse setting. Note that when categorical covariates are present, the summation over *j*, *k* in the regression model in (1) is adjusted such that interactions between their levels are excluded.

### Alternative linked shrinkage models

We discuss a few variations of Bayint (1) that may be relevant for other settings or foci. First, Bay0int, which does not apply shrinkage to the main main effects (non-informative Gaussian priors). This may be useful when one thinks of our model as (a simplification of) a general quadratic form, for which shrinkage of the main effects towards 0 is not necessarily logical. A potential disadvantage is that one looses the link between shrinkage of the two types of effects. Second, Bayintadd, which replaces *τ*_*j*_ *τ*_*k*_ by $\left(\tau_{j}^{2}+\tau_{k}^{2}\right)/2$ , which lets the strongest main effect dominate the shrinkage of the interaction. That is, if any of the two main effects is strong, this leads to relatively little shrinkage (large prior variance) of *β*_*jk*_.

If one is particularly interested in detecting interactions, a third alternative may be attractive: Bayint*, which sets *τ*_int_=1. This model does usually not compete with Bayint in terms of prediction accuracy, as the latter has a global shrinkage parameter *τ*_int_ that can adapt to the interactions being weaker (on average) than the main effects for most problems. The downside of including *τ*_int_, though, is that relatively strong interactions may be over-shrunken, which is why Bayint* may be better at detecting those. Comparisons with these alternative models are provided further on.

## Results

This section consists of three main parts. First, we assess model (1) (i.e. Bayint) in a broad sense by considering parameter estimation, prediction and variable selection. For that, we compare Bayint to the competitors discussed in the Introduction, first for simulated data. Then, the resulting top-5 methods are evaluated in a real data setting for which we may assume (nearly) true values to be known. When relevant we also show results for the Bayint alternatives discussed above. Second, we show how to interpret and infer results from our model using a novel formula for the Shapley values. Finally, for the data, predictive accuracy of Bayint is also compared to that of a non-regression based learner, the random forest. Implementation details of the various methods are provided in the Supplementary Material.

### Evaluation criteria

We evaluate Bayint and its competitors on the basis of the following criteria:

1. Parameter estimation. The root Mean Squared Error (rMSE), defined by
$$\text{rMSE}=\sqrt{\frac{1}{B}\overset{B}{\underset{b=1}{\sum }}{\left({\hat{\beta }}^{\left(b\right)}-\beta \right)}^{2}},$$
  with *β*=*β*_*j*_ or *β*=*β*_*jk*_ and ${\hat{\beta }}^{\left(b\right)}$ the estimator of *β* for the *b*th training set.
2. Prediction. Mean Squared Error of the predictions:
$$\text{MSEp}=\frac{1}{B}\overset{B}{\underset{b=1}{\sum }}\|X_{\text{test}}{\hat{\beta }}^{\left(b\right)}-X_{\text{test}}\beta {\|}_{2}^{2},$$
  with *X*_test_: design matrix for large test sample, including all two-way interactions.
3. Variable selection. Sensitivity at prescribed False Discovery Rate (FDR) levels

Here, the second criterion is equivalent to evaluating prediction error, which includes the noise, but provides a more direct comparison of the fit with the true linear predictor.

### Simulations

We consider two simulation settings, which we summarize here. For the first, we generated *p*=10 moderately collinear continuous covariates for *n*=200 samples, rendering *q*=*p*(*p* − 1)/2=45 two-way interactions. Five main effects are non-zero. For the non-zero interactions, we aimed for a realistic balance of three different types of interactions: six interactions of two non-zero main effects, four interactions between a non-zero and an absent main effect, and finally, three ‘surprising’ interactions between two absent main effects. Two of these interactions have one covariate in common. This implies 18 non-zero coefficients in total. These coefficients range from small to large for main effects, and from small to medium for interaction effects. The second simulation increases sample size to *n*=500 and adds four noise variables, rendering *p*=14 and *q*=91. We kept the same structure for the present main and interaction effects, but given that this setting is sparser than the first, we increased the signal for one main effect and one of each of the three interaction types. For generating the linear response, Gaussian noise was added such that *R* ^2^≈0.5. For both settings, *B*=50 datasets were generated. Details of the simulations are provided in the Supplementary Material.

Table 1 summarizes the performances of the methods on parameter estimation, prediction and selection.

**Table 1:** Summarized performance for simulation 1 (*n*=200, *p* + *q*=55, 18 non-zero effects) and simulation 2 (sparser; *n*=500, *p* + *q*=105, 18 non-zero effects).

Method	Simulation 1			Simulation 2		
	Estimation	Prediction	Selection	Estimation	Prediction	Selection
Bayint	++	++	++	++	++	++
hlasso	++	++	+−	++	++	+−
adlasso	+	+−	+	++	+	+
SandS	+−	+	+	+−	+	+
HS	+	+−	+	+	–	+
lassoint	+	++	^a^	+	+	^a^
ridge2	++	+	^a^	–	–	^a^
OLS	–	−−	+−	–	−−	+−
2-step	−−	−−	^a^	−−	−−	^a^

^a^Method not suitable for selection.

An extensive discussion of the results is included in the Supplementary Material; here we focus on the top-5 methods. Supplementary Figures 1 and 2 show that, generally, Bayint performs very well on estimating the non-zero interactions. Even for the two interactions that are not linked to any non-zero main effects, but do share one covariate, the linked shrinkage provides very competitive performance. The slightly worse estimation of hlasso of the non-zero interactions and large main effects is counterbalanced by better shrinkage for the true zero interactions. The adaptive lasso, adlasso, is quite competitive to hlasso, in particular for the larger sample size (Simulation 2) for which the OLS penalty weights stabilize. The spike-and-slab, SandS, compresses the true zero effects well, but this comes at the prize of relatively unstable estimation of the non-zero effects. The horseshoe, HS, estimates the latter better, but at the price of inferior compression of the non-zero interaction effects.

For prediction, Bayint and hlasso are very competitive to one another, somewhat better than adlasso and SandS, and markedly better than HS as shown in Supplementary Figure 3.

Finally, selection was evaluated by considering sensitivity at fixed FDR levels=0.1, 0.2 (Supplementary Table 1). Here, Bayint outperforms the others, although adlasso, SandS and HS are competitive runner-ups. We noted that the gain in sensitivity for Bayint was not exclusive for interactions; also the main effects were detected better. This is likely due to the local shrinkage and ‘reverse borrowing’ in our model, as the shrinkage of main effects also learns from the linked interactions. The default hlasso did not perform well. This is due to its property of forcing in main effects for selected interactions, which renders too many false positives; countering this by a more stringent penalty compromises the sensitivity. We also present an alternative strategy, which simply sets small effect sizes (≤0.05) to zero. This improves results of hlasso, but still not to the level of the other methods.

### Data

For the data, we present the results of the methods that performed most competitively in the Simulations (top 5): our model (Bayint), adaptive lasso (adlasso), hierarchical lasso (hlasso), spike-and-slab (SandS) and horseshoe (HS).

The main data we use throughout the manuscript is obtained from the Helius study [13]. We use this data set, because it reflects a fairly standard epidemiological study and contains a mix of binary, continuous and categorical covariates. We consider both systolic blood pressure (log scale; SBP) and cholesterol as response, and age, gender, ethnicity (5 levels; coded with 4 dummies), smoking (yes/no), packyears, coffee consumption (yes/no), BMI and 4 simulated standard normal noise variables as covariates, rendering *p*=14 covariates. All two-way interactions are considered, except those between the 4 dummy variables representing the categorical covariate, rendering q=142−42=91−6=85 interaction parameters.

The entire data set, referred to as the ‘master set’, consists of n=21,570 samples. Therefore, OLS estimates based on the master set are very precise, and hence safely used as benchmarks. As a verification, we confirm that i) the estimated coefficients of the noise variables are indeed very close to zero; and ii) the coefficients estimated by (adaptive) lasso are very close to the OLS estimates (Supplementary Figures 4 and 5).

Continuous covariates were centered and scaled, that is standardized. On the centering, we follow the advise by Afshartous and Preston [1]; as the centering (largely) removes collinearity between main effects and two-way interactions. Scaling is generally applied in shrinkage settings, and also helps to interpret the coefficients and estimation errors relative to one another. Binary covariates were (contrast) coded as −1, 1, which renders them standardized in the balanced setting. For interpretation, we prefer to use the same coding for all binaries. The categorical covariate, ethnicity, was contrast-coded with levels −1, 0, 1.

#### Parameter estimation

**Figure 1:**
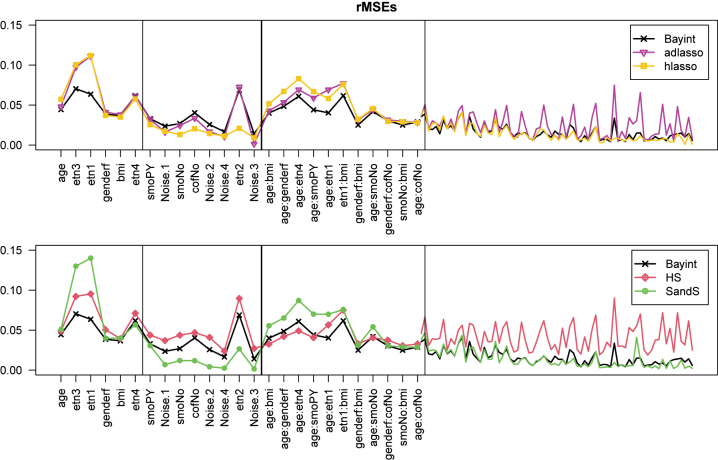
rMSEs for 14 main effects and 85 interactions (before and after bold vertical line), each ordered by significance in master set. Thin vertical line demarcates strong and weak effects in the master set (criterion: *p* < 0.01). **Cholesterol** as outcome. Spacing for weak interactions adjusted to 1/4th for visual purposes.

**Figure 2:**
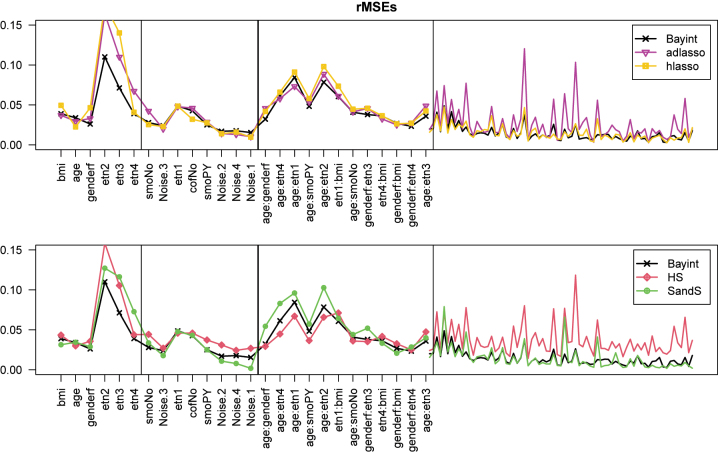
rMSEs for 14 main effects and 85 interactions (before and after bold vertical line), each ordered by significance in master set. Thin vertical line demarcates strong and weak effects in the master set (criterion: *p* < 0.01). **SBP** as outcome. Spacing for weak interactions adjusted to 1/4th for visual purposes.

We use the rMSE as defined above to evaluate parameter estimation. For this, we divided the master set in *B*=25 (nearly non-overlapping) training sets of size *n*=1,000, and set the ‘true’ *β* to the OLS estimate from the large master set. For Bayesian methods, the posterior mean was used as a point estimate for *β*. Figures 1 and 2 compares the results of Bayint with other methods (see Introduction) for cholesterol and SBP as outcome, respectively. The bold line demarcates the main effects and interactions; the thin lines separate the strong effects from the weaker ones, as defined by significance in the master set (*p*<0.01).

Overall, we observe that Bayint shows good estimation performance. For both outcomes, it outperforms the other methods on the estimation of the strong main effects. Hierarchial lasso (hlasso) and spike-and-slab (SandS) compress the weak main effects a bit better than Bayint for the cholesterol outcome. For the strong interaction effects, performances are generally very competitive for the SBP outcome, while Bayint has an edge for the cholesterol, except in comparison to the horseshoe (HS), which is competitive for those effects. The latter, however, lags behind for compressing the weak interaction effects, which is also the case for the adaptive lasso (adlasso). Note that these results are fairly well in line with those from the simulation study.

Supplementary Figure 6 connects the *true* main effects and interactions (estimated from the master set), to provide insight on why linked shrinkage has a benefit for both outcomes. Indeed, we observe that strong interactions tend to link relatively frequently to strong main effects, and that this tendency is somewhat stronger for cholesterol, explaining the slightly larger benefit of linked shrinkage for this outcome compared to SBP.

Finally, we provide a short comparison of Bayint with two aforementioned alternatives, Bay0int and Bayintadd, for the cholesterol model only. From Supplementary Figure 7 we observe that particularly Bayint and Bayintadd are very competitive, with the latter slightly worse for the very non-significant interactions. Bay0int may pick up the strong interactions slightly better, but seems somewhat inferior for main effects and less important interactions.

#### Prediction

**Figure 3:**
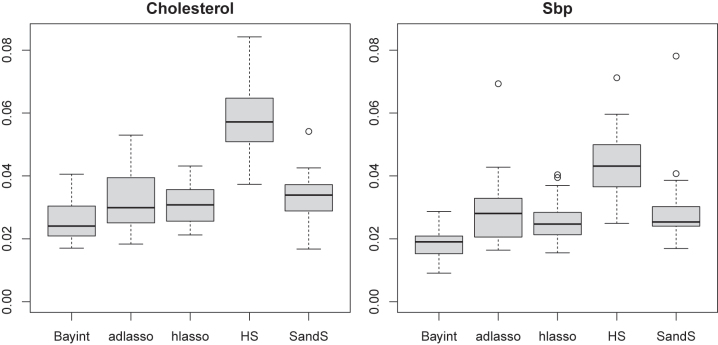
MSE of the predictions of models trained on 25 subsets of the data.

Models were tested on complementary test samples of the master data set. Figure 3 shows the mean squared errors of the predictions (MSEp) for both outcomes. We clearly observe that Bayint, and to a lesser extent, hlasso, outperform the other methods on this criterion. Note here that Bayint reduces the MSEp of hlasso for 22/25 (24/25) subsets and by a relative amount of 17.9 % (26 %) on average for the two outcomes, cholesterol and SBP, respectively.

#### Selection

Next, we focus on selection. Note that the five methods use very different default criteria for performing selection: the lasso-based methods simply report non-zero coefficients for a given penalty parameters, horseshoe and Bayint use credible intervals for a given level, e.g. 95 %, and the spike-and-slab uses a threshold for the posterior selection probability, e.g. 0.5. To be less dependent on such tunable parameters, and allow appropriate comparison, we set these parameters such that FDRs=0.1, 0.2 and compute the sensitivities, as we did for the simulations. For that, we define positives as those significant in the master set at *p*≤0.05/99 (Bonferroni correction) and negatives as those that either correspond to a noise covariate or are non-significant at cut-off 0.05. Given the sheer size of the master set the latter assures that such effects are either very small or completely absent. This defines 12 (14) positives, and 79 (76) negatives for the cholesterol (SBP) outcome; the remaining 99 − 12 − 79=8 (99 − 14 − 76=9) effects are indeterminate, which are therefore not used for calculating the FDRs and sensitivities.

Table 2 shows the results for both outcomes. For the data, the benefit of dedicated shrinkage for the model with all two-way interactions is clear: both Bayint and hlasso outperform the others. Note here that hlasso performance did not improve when applying the extra effect size cut-off discussed in the Simulation section. The gap in performance may be understood by considering the true positive interactions: all of these contained at least one true main effect for both outcomes, which supports linking their shrinkage (or selection) to that of the main effects. For a substantial part of these interactions only one of the two corresponding main effects was a positive. This may explain the better performance of Bayint as compared to hlasso, as the former encodes a softer shrinkage than the latter.

**Table 2:** Sensitivities for variable selection, for fixed FDRs.

FDR	Outcome	Sensitivity				
		Bayint	hlasso	adlasso	HS	SandS
0.10	Chol	**0.490**	0.293	0.180	0.233	0.267
0.20	Chol	**0.583**	0.450	0.283	0.330	0.350
0.10	Sbp	0.451	**0.474**	0.343	0.306	0.394
0.20	Sbp	**0.540**	0.506	0.391	0.343	0.443

The bold values indicate the best performing method for that setting.

For the two strongest methods, we qualitatively compare the actual detections of weak and strong main effects and interactions, where their strengths are defined by the effect sizes in the master data set. Supplementary Figure 8 plots the detections by hlasso for *λ*_min_ and *λ*_1std_ against those by Bayint (using 95 % credible intervals). We observe that the selection by hlasso renders a less clear demarcation of the strong and small effects than Bayint does, in particular for the main effects. Finally, Supplementary Figure 9 compares the detections of Bayint and Bayint*, which applies less shrinkage to the interactions. Indeed, the latter detects strong interactions more often than Bayint, at the cost of detecting two main effects.

### Interpretation of variable importance

Here, we discuss several techniques and visualisations to interpret results from the Bayint model. We focus on cholesterol as outcome. Bayint provides credible intervals. We previously showed the coverage of Bayesian local shrinkage – on which our shrinkage model is based – to be rather good [5] in low dimensional settings, although this will depend on the *p*:*n* ratio and the amount of collinearity. Moreover, Supplementary Table 2 shows that Bayint’s intervals are a powerful tool to select interactions, as compared to other methods. Hence, these intervals are of direct use to infer which interactions are relevant and which are not.

Interpretation and inference for the main effect parameters is hampered though by the presence of those interactions, as the effect of one unit change of a covariate depends on the values of the other covariates. Therefore, technically, *β*_*j*_=0 only means that for a (fictive) person with average values for all other covariates (given centering is applied), covariate *j* has no effect. That is, it only quantifies a *conditional* main effect. Afshartous and Preston [1] propose several useful alternatives, such as determining the ‘range of significance’. For this, one plots the confidence/credible intervals for *β*_*j*_ + *β*_*jk*_ *x*_*ik*_ – the effect of one unit change of *x*_*ij*_ when interacting with one covariate *x*_*ik*_ – against *x*_*ik*_. Alternatively, one may compute *E*_*ij*_=*β*_*j*_ + ∑_*k*≠*j*_ *β*_*jk*_ *x*_*ik*_, i.e. a ‘personalized unit change effect’ which accounts for all interactions. Our MCMC samples easily provide the posteriors of *E*_*ij*_, allowing to plot its uncertainty as well. A hybrid of the latter two solutions is a plot of *E*_*ij*_ against *x*_*ik*_ (when continuous) or for color-coded levels of *x*_*ik*_ to see whether one unit change of *x*_*ij*_ (say age or BMI) has a different effect on the outcome, e.g. for *x*_*ik*_=−1, 1 (say female/male), while accounting for the other interacting covariates as well. Supplementary Figure 10 plots *E*_*ij*_ and its uncertainty for 100 random test individuals (Bayint model fitted on 1,000 training samples), with *x*_*ij*_ and *x*_*ik*_ representing age and gender, respectively. We clearly observe a different effect of age increase between genders, but also within gender due to interactions of age with other covariates.

Alternatively, Shapley values [6] may be considered. A Shapley value *ϕ*_*ij*_ quantifies the average contribution of the *j*th covariate to the prediction of the *i*th sample, fixing $x_{ij}=x_{ij}^{*}$ . Here, ‘average’ refers to a weighted average over subsets S that contains all other covariates ${\left(x_{ik}\right)}_{k\in S}$ that actively impact the prediction by fixing $x_{ik}=x_{ik}^{*}$ (called the ‘players’). Predictions are marginalized over the complement, $S^{\prime }$ , which defines the non-players ${\left(x_{i\ell }\right)}_{\ell \in S^{\prime }}$ , which are considered random. Here, the weights are chosen such that different sizes of S have an equal impact on the Shapley value. A formal definition is given in the Appendix. Inference based on Shapley values is a useful complement to parameter inference: the first is global in the sense of a covariate’s importance, whereas the second is local in that respect; the reverse is true for samples: the first is sample-specific, hence local, whereas a parameter is shared by all samples, hence global.

**Figure 4:**
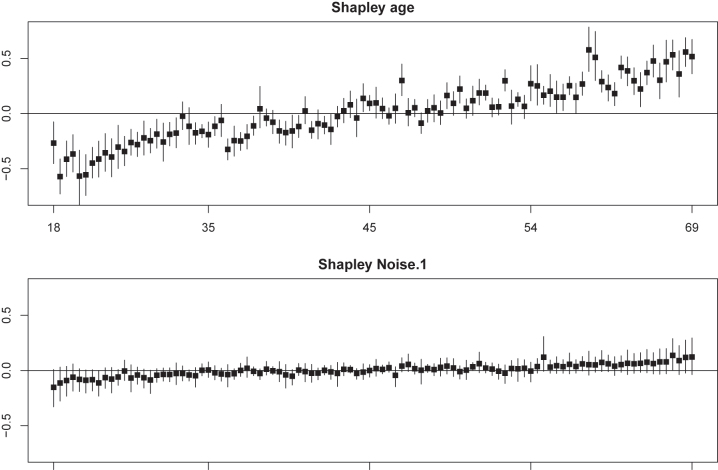
Shapley values of ‘age’ and ‘Noise.1’ and their posteriors for 100 random test individuals, ordered by ‘age’ (original scale) and ‘Noise.1’, respectively. **Cholesterol** (standardized) as outcome.

Shapley values are popular in machine learning nowadays, because they uniquely possess several nice properties: efficiency, symmetry, dummy player and linearity [6]. Obtaining its exact value is usually computationally very demanding, let alone computing uncertainties. For our model, however, it is feasible to compute Shapley values and their uncertainties efficiently, if one is willing to use the common convention that the marginalization ignores the dependency between the players and the non-players [14], an approach referred to as ‘interventional Shapley value’ [6]. For a linear regression model with two-way interactions and centered covariates it equals

(2) $$\phi_{ij}=\beta_{j}x_{ij}^{*}+\frac{1}{2}\left(\underset{k:k\neq j}{\sum }\beta_{jk}x_{ij}^{*}x_{ik}^{*}-\underset{k:k\neq j}{\sum }\beta_{jk}E\left[x_{ij}x_{ik}\right]\right),$$

when the *j*th covariate is continuous or binary. A proof is provided in the Appendix, which also includes expressions for the non-centered setting and categorical covariates. Note that (the posterior of) *ϕ*_*ij*_ is straightforward to compute after estimating *E*[*x*_*ij*_ *x*_*ik*_] by the sample covariance. Again, we illustrate results for 100 random test samples and the Bayint model trained on 1,000 random training samples. Figure 4 shows Shapley values and their credible intervals for ‘age’ and ‘Noise.1’. The latter is a useful negative control as we observe that, as desired, all credible intervals cover 0. Note that centering of the covariates implies that Shapley values are expected to center around 0. Yet, we observe that age is an important covariate for the majority of samples as most intervals do not cover 0. The plot for ‘age’ also shows that interactions are not equally important for all samples: those that deviate relatively much from the trend, like the first one, are impacted more due to the $x_{ij}^{*}x_{ik}^{*}$ term in (2). Supplementary Figures 13 and 14 provide empirical evidence that the intervals, as computed from the output of Bayint, provide appropriate coverage for the majority of covariates and individuals.

**Figure 5:**
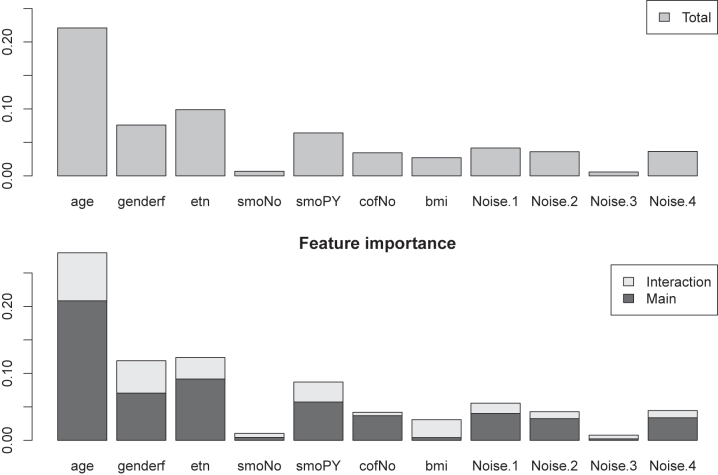
Global variable importance scores. Top: mean absolute Shapley values (*I*_*j*_); Bottom: mean absolute contributions of main effect $\left(I_{j}^{\text{main}}\right)$ and interactions $\left(I_{j}^{\text{int}}\right)$ . **Cholesterol** as outcome.

Clearly, $\phi_{ij}=\phi_{ij}^{\text{main}}+\phi_{ij}^{\text{int}}$ , which denote the contributions of the main effect and that of all interactions with covariate *j*. Supplementary Figure 15 displays the Shapley values (posterior means), and its two contributors, for all covariates. Alternatively, Figure 5 shows the conventional variable importance derived from Shapley values, $I_{j}=1/n\sum_{i=1}^{n}|\phi_{ij}|$ , and analogously defined, $I_{j}^{\text{main}}$ and $I_{j}^{\text{int}}$ . Note that, in general, $I_{j}\neq I_{j}^{\text{main}}+I_{j}^{\text{int}}$ . Still, plotting both $I_{j}^{\text{main}}$ and $I_{j}^{\text{int}}$ renders insight on how relevant the main effects and interactions are for each covariate. While the main effects show the strongest importance scores for most covariates (except BMI), we do observe that interactions are also relevant for a fair share of the covariates.

Finally, we compared the estimated Shapley values with the ‘true ones’, which are available by substituting the coefficients from the large master set into (2). We did so for both our method, Bayint, and the hierarchical lasso, hlasso. Supplementary Figure 16 shows that both methods estimate the Shapley values well for ‘age’, which is a strong, continuous covariate. For a weaker, binary covariate, ‘etn1’, we observe that Bayint provides much better estimation of the Shapley values than hlasso. This is in line with the results in Figure 1: Bayint has smaller estimation errors of the parameters in which this covariate is involved. Finally, a more nuanced difference in performance is visual for the covariate ‘gender’ in Supplementary Figure 18, which shows a benefit of Bayint for some training sets, but not all.

### Model assessment by *R* ^2^

So far we have compared our method Bayint with competitors that use the exact same model (regression with all two-way interactions), but different types of regularizations. Here, we broaden the scope and compare the overall out-of-sample fit of Bayint with a basic regression model without interactions as well as with a machine learner, here the random forest. The first comparison allows to judge the additive value of the interactions for improving test sample fit, whereas the second one is relevant, because the random forest holds the promise to capture interactions well and to provide adequate predictions, so it provides a useful benchmark. As an additional benchmark, we also include OLS with all two-way interactions in the comparison.

Random forest was either fit using the defaults in the randomForestSRC package (RF) or with hyperparameters (mtry: number of features considered per split and nodesize: minimum node size) tuned for optimal predictive performance using the tune.rfsrc function (RFtune).

**Figure 6:**
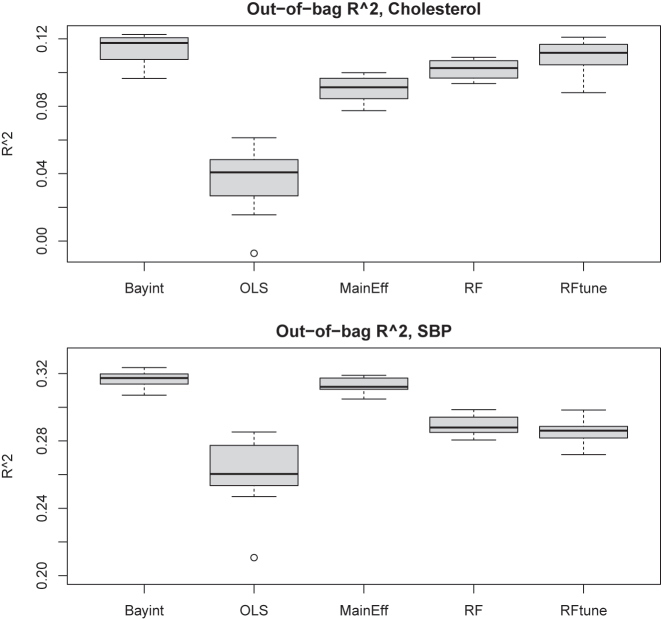
Out-of-bag *R* ^2^s for 25 training sets of size *n*=1,000 for cholesterol (top) and SBP (bottom) as outcome. Methods: Bayint: Bayesian linked shrinkage model; OLS: OLS with all terms; MainEff: OLS with main effects only; RF (RFtune): Random forest with default (tuned) parameters.

For the comparison, we compute for each model and for all *b*=1, …, 25 training sets the out-of-bag coefficient of determination, $R_{b}^{2}=1-\sum_{i\in T_{b}}{\left(y_{i}-{\hat{y}}_{i,b}\right)}^{2})/\sum_{i}{\left(y_{i}-\bar{y}\right)}^{2}$ , with $T_{b}$ the set of all out-of-bag samples for training *b*, and ${\hat{y}}_{i,b}$ the prediction for test sample *i* by the *b*th model. Figure 6 shows the results.

For cholesterol as outcome, we observe that Bayint provides a substantial gain in terms of *R* ^2^ as compared to its OLS counterpart, and a moderate improvement w.r.t. the main effects only model. Moreover, predictive performance is somewhat better than that of RF, and marginally better than that of RFtune. The latter two overfit substantially, as observed from comparing the in-bag and out-of-bag performances (Supplementary Figure 11). For SBP observe that all models predict this outcome than cholesterol. Here, differences between Bayint and the main effects only model are small, whereas both beat OLS, RF and RFtune by a fair margin. Note that the relative performance of the random forest improves for *n*=5,000 (Supplementary Figure 12), rendering it competitive to Bayint in terms of prediction.

Hence, from a predictive perspective Bayint clearly outperforms its OLS counterpart. Moreover, it is highly competitive, if not superior, to a much simpler model, the main effects only model, which provides no information on interactions, and to a more complex one, RF, the results of which are more difficult to interpret and infer.

## Implementation and data availability

Our linked shrinkage model was implemented in RStan (v 2.21.8) [15]. We chose to use a general purpose sampler for several reasons. First, it allows the user to adjust the model without much extra effort in terms of fitting or inference. This includes variations on modelling the shrinkage, as illustrated, but also adjusting the likelihood to allow binary or survival outcome, as RStan accommodates these as well. We provide an example for logistic regression in the code. Second, RStan provides several diagnostic tools, such as trace plots, to check the convergence of the MCMC sampler. Our scripts are available at https://github.com/markvdwiel/ThinkInteractions/, which also contains a synthetic version of the data set. The covariates in the synthetic data are generated by imputation as described in van de Wiel et al. [5]. Then, responses (Cholesterol and SBP) are generated by drawing from normal distributions with means equal to the OLS (as fitted on the master set) predictions based on the synthetic covariates, and error variance equal to the residual error variance of the OLS model. We verified that the synthetic set renders qualitatively similar results on the regression models as those presented here (cf. Figure 1 and Supplementary Figure 19; Figure 2 and Supplementary Figure 20). Finally, as an indication: running time for our example data sets of *n*=1,000, *p*=14, *q*=85 is around 3 min for 25,000 MCMC samples using a single core from a PC with a 1.30 GHz processor and 16 Gb RAM.

## Discussion

We demonstrated the potential of linked shrinkage for improving parameter estimation in fairly large regression models that include all two-way interactions. Naturally, the benefit depends on the data set and the relevance of those interactions, in particular in connection to the main effects. A limitation of our main model, Bayint, is that it may not perform well for interactions for which none of the main effects are relevant. We showed, however, that when such interactions share a common covariate, their linked shrinkage offers a benefit, rendering very competitive performance. Moreover, we offer an alternative, Bay0int, which provides more power to detect ‘surprising’ interactions at the cost decreased performance for the main effects, as compared to Bayint. Another limitation might be the linear scale of the covariates in the model. As regression comes with many tools for model diagnostics, such a model miss-specification can be diagnosed. In principle, it is straightforward to extend our model by adding non-linear transformations (log, quadratic) of a few covariates, each with their own local penalty. Optimizing the model by including such covariate transformations after viewing the data may come at the cost of invalid inference. Possibly, for large *n*, the cost of this form of data snooping may be limited when only the main effects are considered, but this requires further study. In addition, note also that if the model is very wrong its predictive performance is likely compromised, as compared to more flexible machine learners. This was not the case for our data, at least in comparison to the random forest.

We showed that Bayint is a very viable alternative for its main competitor, hlasso: it performs competitively, if not slightly better, on parameter estimation and prediction, while providing the extra benefit of uncertainty quantification. Moreover, it outperforms hlasso in terms of selection. Selection instability is a well-known problem for the lasso, which may be countered by applying stability selection [16]. This requires multiple fits for bootstrapped samples. If selection is the sole aim of the study – as is often the case in high-dimensional settings – this is a very useful solution. In our setting, however, we strived for a method that provides estimation, prediction, selection, uncertainty quantification and interpretation with one single model fit.

A natural extension of our method is the inclusion of higher-order interaction. In principle, this is easily achieved with our code, and may be a viable option when only few covariates are present. Note, however, that the number of three-way interactions increases cubicly with the number of covariates, and one may argue that regression models with higher-order interaction are hardly any easier to interpret than many machine learners.

As mentioned, we coded our method in RStan, because its flexibility allows the user to extend the framework to one’s own needs, such as the various illustrated shrinkage links and non-continuous outcomes (survival, binary). A potential disadvantage of using such a general purpose sampler is that it is likely too slow for large *p*, large *n* settings. RStan provides variational Bayes approximations, but we experienced that both the mean-field and the (Gaussian) full-rank approximations do not provide satisfactory results (compared to sampling) for our model. Alternatively, the RStan manual suggests to use a thin QR-decomposition of the design matrix for large scale regression problems, but this did not speed up computations in our setting, probably due to the non-exchangeable prior for the regression coefficients. Dedicated sampling or variational Bayes techniques such as proposed for the horseshoe prior in Makalic and Schmidt [17] and Busatto and van de Wiel [18] may provide more scalable alternatives, but this requires more research.

While certainly simpler than many machine learners, the interpretation of a regression model with many two-way interactions is not trivial [1]. Inference for parameters and variable importance scores aids such interpretation. Therefore, we showed that our methods provides a good basis for inference, which we extend to variable importance scores, in particular the Shapley value. We derived the computational efficient formula (2) for its interventional version. If one prefers to account for dependency when marginalizing, approaches as in Aas et al. [6] may be explored, but likely at a high computational cost. For other regressions, e.g. logistic or Cox, formula (2) is only valid if one evaluates the prediction on the level of the linear predictor. This may be reasonable as the regression coefficients are also on that scale.

While it is straightforward to define global variable importance scores from the personalized ones, including Shapley, it less obvious how to perform inference for these, both from a technical and philosophical perspective. As for the first: one may average absolute or squared scores, but the result lacks a natural null. As for the second: in such models, the importance of a covariate depends on the values of the other ones, and is hence individual specific. Possibly, an informal argument, such as: the credible intervals should not contain ‘0’ for at 10 % of the individuals, may be reasonable in practice, but this needs further research.

Finally, we illustrated that the addition of two-way interactions, as in Bayint, may improve predictive performance with respect to a main effect only regression model. In fact, Bayint can be very competitive to more advance machine learners, such as random forest. Of course, such comparative results depend strongly on the data, sample size and true complexity of the associations between covariates and response. If prediction is an important aim of the study, possibly alongside interpretation, we recommend comparing the predictive performance of Bayint with more flexible machine learners. In the eventual case of inferior predictive performance of the former, one should balance this loss against the improved interpretability.

All-in-all, Bayint is an attractive alternative for existing strategies to handle interactions in epidemiological and/or clinical studies, as its linked local shrinkage can improve parameter accuracy, prediction and variable selection. Moreover, it provides appropriate inference and interpretation, and may compete well with less interpretable machine learners in terms of prediction.

## Supplementary Material

Supplementary Material Details

## Appendix: Shapley values

Here, we derive the interventional Shapley value for our model. For clarity we fix the sample index *i*, and denote *x*_*j*_=*x*_*ij*_ and its realization by $x_{j}^{*}$ . W.l.o.g. we assume index *p* for the covariate of which we compute the Shapley value. Denote by S⊆P={1,…,p} the set of players, i.e. the covariate indices for which their value is set at the realization, and its complement $S^{\prime }$ : the set of non-players over which the predictions are marginalized. Define weights w(S)=1pp−1|S|−1 . Write $x={\left(x_{j}\right)}_{j=1}^{p},x_{S}={\left(x_{j}\right)}_{j\in S},x_{S}^{*}={\left(x_{j}^{*}\right)}_{j\in S}$ , and *f*(***x***) for the predicted value. Moreover, let $\nu \left(S\right)=E\left[f\left(x\right)|x_{S}=x_{S}^{*}\right]$ , which is the expected predicted value, with the expectation computed over all covariates $j\in S^{\prime }$ . Then,

(3)
$$\phi_{p}=\underset{S\in P\backslash \left\{p\right\}}{\sum }w\left(S\right)\left(\nu \left(S\cup \left\{p\right\}\right)-\nu \left(S\right)\right).$$

For the interventional Shapley, it is assumed that $P\left(x_{S^{\prime }}|x_{S}=x_{S}^{*}\right)=P\left(x_{S^{\prime }}\right)$ . Then, $\nu \left(S\right)=E_{x_{S^{\prime }}}\left[f\left(x_{S}^{*},x_{S^{\prime }}\right)\right]$ .

The following result shows that for a linear regression model with all main effects and two-way interactions the exact *ϕ*_*p*_ can be computed with linear complexity O(p) instead of exponential. For simplicity, we first assume categorical variables are absent.

Theorem 1. Let *ϕ*_*p*_ be defined as in (3) and assume $P\left(x_{S^{\prime }}|x_{S}=x_{S}^{*}\right)=P\left(x_{S^{\prime }}\right)$ . Then for $f\left(x\right)=\alpha_{0}+\sum_{j=1}^{p}\beta_{j}x_{j}+\sum_{k=2}^{p}\sum_{j=1}^{k-1}\beta_{jk}x_{j}x_{k}$ we have:

(4)
$$\phi_{p}=\beta_{p}\left(x_{p}^{*}-E\left[x_{p}\right]\right)+\frac{1}{2}\left(\overset{p-1}{\underset{j=1}{\sum }}\beta_{jp}\left(E\left[x_{j}\right]x_{p}^{*}-E\left[x_{j}x_{p}\right]\right)+\overset{p-1}{\underset{j=1}{\sum }}\beta_{jp}\left(x_{j}^{*}x_{p}^{*}-x_{j}^{*}E\left[x_{p}\right]\right)\right).$$

Proof. First, for convenience we write $\sum_{k=2}^{p}\sum_{j=1}^{k-1}\beta_{jk}x_{j}x_{k}=\frac{1}{2}\sum_{j,k}\beta_{jk}x_{j}x_{k}$ , with *β*_*kj*_=*β*_*jk*_ and *β*_*jj*_=0. Then, decompose ν(S) into the main effects term (including the intercept) and an interaction term: $\nu \left(S\right)=\nu_{m}\left(S\right)+\nu_{i}\left(S\right)$ . Analogously, write $\phi_{p}=\phi_{p}^{m}+\phi_{p}^{i}$ . Aas et al. [6] show that $\phi_{p}^{m}=\beta_{p}\left(x_{p}^{*}-E\left[x_{p}\right]\right)$ . Hence, we focus on the contribution of the interactions, which equals:

$$\nu_{i}\left(S\right)=\frac{1}{2}\left(\underset{\begin{array}{c} j\in S^{\prime }\\ k\in S^{\prime } \end{array}}{\sum }\beta_{jk}E\left[x_{j}x_{k}\right]+2\underset{\begin{array}{c} j\in S\\ k\in S^{\prime } \end{array}}{\sum }\beta_{jk}x_{j}^{*}E\left[x_{k}\right]+\underset{\begin{array}{c} j\in S\\ k\in S \end{array}}{\sum }\beta_{jk}x_{j}^{*}x_{k}^{*}\right).$$

Likewise,

$$\nu_{i}\left(S\cup \left\{p\right\}\right)=\frac{1}{2}\left(\underset{\begin{array}{c} j\in S^{\prime }\backslash \left\{p\right\}\\ k\in S^{\prime }\backslash \left\{p\right\} \end{array}}{\sum }\beta_{jk}E\left[x_{j}x_{k}\right]+2\underset{\begin{array}{c} j\in S\cup \left\{p\right\}\\ k\in S^{\prime }\backslash \left\{p\right\} \end{array}}{\sum }\beta_{jk}x_{j}^{*}E\left[x_{k}\right]+\underset{\begin{array}{c} j\in S\cup \left\{p\right\}\\ k\in S\cup \left\{p\right\} \end{array}}{\sum }\beta_{jk}x_{j}^{*}x_{k}^{*}\right).$$

Therefore, we have:

(5)
$$\begin{array}{cc} \nu_{i}\left(S\cup \left\{p\right\}\right)-\nu_{i}\left(S\right) & =-\underset{j\in S^{\prime }\backslash \left\{p\right\}}{\sum }\beta_{jp}E\left[x_{j}x_{p}\right]+\underset{j\in S^{\prime }\backslash \left\{p\right\}}{\sum }\beta_{jp}E\left[x_{j}\right]x_{p}^{*}-\underset{j\in S}{\sum }\beta_{jp}x_{j}^{*}E\left[x_{p}\right]+\underset{j\in S}{\sum }\beta_{jp}x_{j}^{*}x_{p}^{*}\\ & =\underset{j\in S^{\prime }\backslash \left\{p\right\}}{\sum }T_{j}^{1}+\underset{j\in S}{\sum }T_{j}^{2}, \end{array}$$

with $T_{j}^{1}=\beta_{jp}\left(E\left[x_{j}\right]x_{p}^{*}-E\left[x_{j}x_{p}\right]\right)$ and $T_{j}^{2}=\beta_{jp}\left(x_{j}^{*}x_{p}^{*}-x_{j}^{*}E\left[x_{p}\right]\right)$ . Then,

$$\phi_{p}^{i}=\underset{S}{\sum }w\left(S\right)\left(\underset{j\in S^{\prime }\backslash \left\{p\right\}}{\sum }T_{j}^{1}+\underset{j\in S}{\sum }T_{j}^{2}\right).$$

To compute $\phi_{p}^{i}$ , we have for the second term:

∑Sw(S)∑j∈STj2=∑Sw(S)∑j=1p−1I(j∈S)Tj2=∑k=1p−1wℓp−2k−1∑j=1p−1Tj2,

as the weight w(S)=wℓ=1/p[p−1ℓ]−1 , when |S|=ℓ , and p−2ℓ−1 counts the number of size *ℓ* subsets out of {1, …, *p* − 1} that necessarily contain *j*, which equals the number of size *ℓ* − 1 subsets out of {1, …, *p* − 1}\{*j*}. Likewise for the first term:

∑Sw(S)∑j∈S′\{p}Tj1=∑Sw(S)∑j=1p−1I(j∈S′\{p})Tj1=∑ℓ=0p−2wℓp−2p−2−ℓ∑j=1p−1Tj1,

as |S|=ℓ implies $|S^{\prime }\backslash \left\{p\right\}|=p-1-\ell$ and p−2p−2−ℓ counts the number of size *p* − 1 − *ℓ* subsets out of {1, …, *p* − 1} that necessarily contain *j*. Finally, note that wℓp−2ℓ−1=(1/p)p−2ℓ−1/p−1ℓ=ℓp(p−1) , and wℓp−2p−2−ℓ=p−1−ℓp(p−1) and that $\sum_{\ell =0}^{p-2}\left(p-2-\ell \right)=\sum_{\ell =1}^{p-1}\ell =p\left(p-1\right)/2$ . Substitution completes the proof. □

Corrollary 1. If covariates are centered, we have:

(6)
$$\phi_{p}=\beta_{p}x_{p}^{*}+\frac{1}{2}\left(\overset{p-1}{\underset{j=1}{\sum }}\beta_{jp}x_{j}^{*}x_{p}^{*}-\overset{p-1}{\underset{j=1}{\sum }}\beta_{jp}E\left[x_{j}x_{p}\right]\right).$$

Proof. This simply follows from the fact that centering implies *E*[*x*_*j*_] = 0. □

Corrollary 2. If a categorical covariate is present, then:

1. For a non-categorical covariate *ϕ*_*p*_ remains unchanged
2. For a categorical covariate $\tilde{p}$ with *Q* levels, corresponding to indices {*p* + 1 − *Q*, …, *p*},
  (7)
  $$\phi_{\tilde{p}}=\overset{Q}{\underset{q=1}{\sum }}\phi_{p+1-q}.$$

Proof. First, *1.* follows directly from redefining $P=1,\ldots ,p^{-}$ , where *p* ^−^ is the number of covariates, counting categorical variables as one. When a categorical variable $\tilde{p}$ is in S all its modeling components contribute to ν(S) . Then, replacing *p* by *p* ^−^ in the weights *w* and in the combinatorial argument above, renders exactly the same result for a non-categorical covariate. For *2.*, simply note that $\nu \left(S\cup \left\{\tilde{p}\right\}\right)$ extends linearly over all regression terms with indices *p* + 1 − *Q*, …, *p*, when excluding interaction terms between levels of the $\tilde{p}$ in the regression model, as is the convention. □
