# Supplementary material for: Linked shrinkage to improve estimation of interaction effects in regression models
Source: Epidemiol Methods. 2024 Jul 9;13(1):20230039. doi: 10.1515/em-2023-0039 (PMC11232106; doi:10.1515/em-2023-0039)
Supplement: Supplementary file 1 — Supplementary Material Details [file j_em-2023-0039_suppl_001.pdf]

SUPPLEMENTARY MATERIAL to: "Linked shrinkage to improve estimation of interaction effects in regression models"

1 Implementation of alternative methods

Below we give details on the software used for each of the alternative methods used in the manuscript. Code is available on: `https://github.com/markvdwiel/ThinkInteractions/`.

- `OLS` and `2step` are fit using the base `R lm` function, where the latter first fits a 'main effect only model', and then fits a new model with only the significant main effects ($p < 0.05$) and their interactions
- `ridge2` is fit using `mgcv` (v 1.8-42), which automatically tunes the two penalties
- `lassoint` and `adlasso` are fit using `glmnet` (v 4.1-7) using the penalty that minimises the 10-fold cross-validated squared prediction error (default). Penalty weights for `adlasso` are the reciprocal absolute `OLS` coefficients
- `hlasso` is fit using `glinternet` (v 1.0.12) with the penalty cross-validated as for `lassoint` (default)
- `SandS`, the spike-and-slab model, is fit using `R2BGLiMS` (v 0.1), with default settings
- `hs`, the horseshoe model, is fit using `horseshoe` (v 0.2.0), with default settings
- Variations of our model, `Bayintadd`, `Bay0int` and `Bayint*`, as well as `Bayloc` are implemented in `RStan` (v 2.21.8), using the exact same priors and sampling scheme as used for our model (`Bayint`)
- `RF` is either fit using the defaults of the `rfsrc` function in the `randomForestSRC` package (v 3.2.2) or with hyperparameters (`mtry` and `nodesize`) tuned for optimal predictive performance using the `tune.rfsrc` function

2 Simulation results

2.1 Set-up

We simulated two settings, described below.

Simulation 1

- $n = 200$
- $p = 10$. All two-way interactions are considered.
- All two-way interactions are considered. So, $q = p(p-1)/2 = 45$.
- Main effects: $\beta_1 = 0.5, \beta_2 = -0.4, \beta_3 = 0.3, \beta_4 = 0.2, \beta_5 = 0.1$. $(\beta_j)_{j=6}^{10} = \mathbf{0}$
- Six non-zero interactions corresponding to **two** non-zero main effects:
$\beta_{12} = 0.3, \beta_{13} = 0.2, \beta_{15} = -0.1, \beta_{23} = -0.1, \beta_{24} = 0.3, \beta_{34} = 0.2$

- Four non-zero interactions corresponding to **one** non-zero main effect:
  $\beta_{16} = 0.3, \beta_{19} = 0.2, \beta_{36} = -0.2, \beta_{47} = 0.1$
- Three non-zero interactions corresponding to **no** non-zero main effects:
  $\beta_{67} = 0.3, \beta_{68} = 0.1, \beta_{9,10} = -0.2$
- All 45 - 13 = 32 other interaction parameters β_{jk} are set to 0.
- For $i = 1, \ldots, n$, $X_i = (x_{i1}, \ldots, x_{i10}) \sim \text{MVN}(\mathbf{0}, \Sigma)$, where Σ is covariance matrix with variances equal to 1 and all covariances equals to 0.3.
- $Y_i = \sum_{j=1}^{p} \beta_j x_{ij} + \sum_{j \neq k} \beta_{jk} x_{ij} x_{ik} + \epsilon_i, \epsilon_i = N(0, \sigma^2)$, with σ^2 such that the $R^2 \approx 0.5$ for the true model.

Simulation 2

- $n = 500$
- $p = 14$. All two-way interactions are considered.
- All two-way interactions are considered. So, $q = p(p-1)/2 = 91$.
- $\boldsymbol{\beta}$ generated as for Simulation 1, except for the following twice as strong signals: $\beta_1 = 1, \beta_{12} = 0.6, \beta_{16} = 0.6, \beta_{67} = 0.6$
- 91 - 13 = 78 interaction parameters β_{jk} are set to 0.
- X and Y generated as for Simulation 1

With those simulations we attempted to use settings that may be reasonable in practice: main effects are generally somewhat stronger than interaction effects; interactions are somewhat more likely to be non-zero when one or both corresponding main effects are; some covariates are more likely to have interaction effects than others; some collinearity in the covariates is present. The second simulation is sparser than the first, which is compensated by using a few stronger non-zero signals. Moreover, the two simulations cover different (n, p) settings.

2.2 Criteria

We evaluate the methods on the basis of the following criteria:

- Parameter estimation: rMSEs of the parameters estimates, as defined in the Main document
- Prediction: MSE of the predictions, MSEp, as defined in the Main document
- Variable selection: Sensitivity at prescribed False Discovery Rate (FDR) levels

2.3 Parameter estimation: rMSEs

Figures 1 and 2 show the rMSEs of the parameter estimates for Simulations 1 and 2, respectively, for all methods. Here, we distinguish four categories of parameters: those with true values not-equal or equal to zero, cross-tabulated versus whether they present a main or interaction effect. In addition, the non-zero interactions are divided in three subcategories as indicated above: those corresponding to two, one or zero non-zero main effects. From these results we observe that:

- Spike-and-slab (`SandS`) and `2-step` generally show fairly good compression of the zero effects, but at the high price of inferior estimation for the non-zero effects, in particular for `2-step`.

- `OLS` and horseshoe (`hs`) show a fairly similar pattern, with the horseshoe more competitive to `Bayint`, except for the zero interaction effects, for which both `OLS` and `hs` show substantially larger errors than `Bayint`. That is, the relatively mild shrinkage of `hs` benefits the estimation of the non-zero interaction effects due to its local character, but the lack of structure in the regularization (as compared to `Bayint`) this renders worse estimation of the zero interaction effects.

- `ridge2` shows fairly competitive performance to `Bayint` for Simulation 1, but rather inferior for Simulation 2, in particular for the effects corresponding to large parameter values. Here, the global shrinkage by ridge2 (grouped by the two types of effects, mains and interactions) compresses these parameters too much.

- All lasso variations are rather competitive to one another, with a slight edge for `hlasso` in particular for the small sample size setting, Simulation 1. Naturally, adaptive lasso (`adlasso`) improves for larger n (Simulation setting 2) as the OLS-based penalty weights become more stable. For the non-zero interactions `hlasso` is only slightly better than `lassoint`. Not surprisingly, the latter is somewhat superior for the non-zero main effects, but inferior for the zero main effects, as `lassoint` does not shrink main effects.

- Overall, `hlasso` is quite competitive to `Bayint`. It performs somewhat inferior on estimating the non-zero interactions, and also on estimating the large non-zero main effects (the first two) in particular in Simulation setting 2. The zero interactions, however, are compressed better by `hlasso`.

- For non-zero interactions, we observe that indeed the benefit of linked shrinkage is highest for the left subcategory for which both corresponding main effects are non-zero as well. For the right subcategory - i.e. the surprising interaction (no non-zero main effects) - we observe that `Bayint` still does fairly well for the first two interactions in this subcategory, in particular in Simulation 2. This is likely due to them sharing one covariate (X_6); hence they borrow information from one another. For the last surprising interaction it does, like `hlasso`, perform inferior to many of the ‘unlinked’ methods, as expected.

2.4 Prediction

As Figures 1 and 2 contain results on many parameters, it useful to have an overall assessment of the fit for each of the models. We evaluate this by considering the MSEs of the predictions

(MSEp). Figure 3 show the MSEps of the test sample predictions for both simulation settings for all methods, except 2-step as it rendered a much larger MSEp than the others. We observe that `Bayint` and `hlasso` outperform the others on this matter.

2.5 Selection

Here, we focus on selection. We exclude `ridge2` (not sparse), `lassoint` (does not penalize/select main effects) and `2-step` (unreliable confidence intervals), as these methods are less suitable for this purpose. Note that the other methods use very different default criteria for performing the selections: the lasso based methods simply report non-zero coefficients for a given penalty parameters; OLS, horseshoe and our method use confidence or credible intervals for a given level, e.g. 95%; and the spike-and-slab uses a threshold for the posterior selection probability, e.g. 0.5. To be less dependent on such tunable cut-offs, and allow appropriate comparison, we set these cut-offs such that FDRs = 0.1, 0,2 and compute the sensitivities. Results are shown in Table 1.
We conclude that `Bayint` provides excellent sensitivities at given `FDR` levels, with `adlasso`, `SandS` and `HS` as competitive runner-ups. The default `hlasso` does not perform well. This is due to its property of forcing in main effects for selected interactions, which renders too many false positives for our simulation set-up, as this includes quite a few interactions for which at least one of the terms does not correspond to a non-zero main effect. We also evaluated a strategy that enforces a minimum effect size of 0.05 (`hlasso2`), which indeed performs somewhat better, but still not competitive. Also `OLS` performs inferior to most competitors in those two simulation settings.

FDR	Simulation Setting	Sensitivity						
		`Bayint`	`hlasso`	`hlasso2`	`adlasso`	`HS`	`SandS`	`OLS`
0.10	1	**0.497**	NA*	0.320	0.469	0.471	0.479	0.409
0.20	1	**0.606**	0.417	0.580	**0.603**	0.509	0.578	0.520
0.10	2	**0.573**	0.018	0.327	0.533	0.507	0.516	0.494
0.20	2	**0.663**	0.308	0.578	0.608	0.592	0.603	0.554

Table 1: Sensitivities for variable selection, averaged over $B = 50$ simulations, for fixed FDRs. *: Can not achieve FDR level.

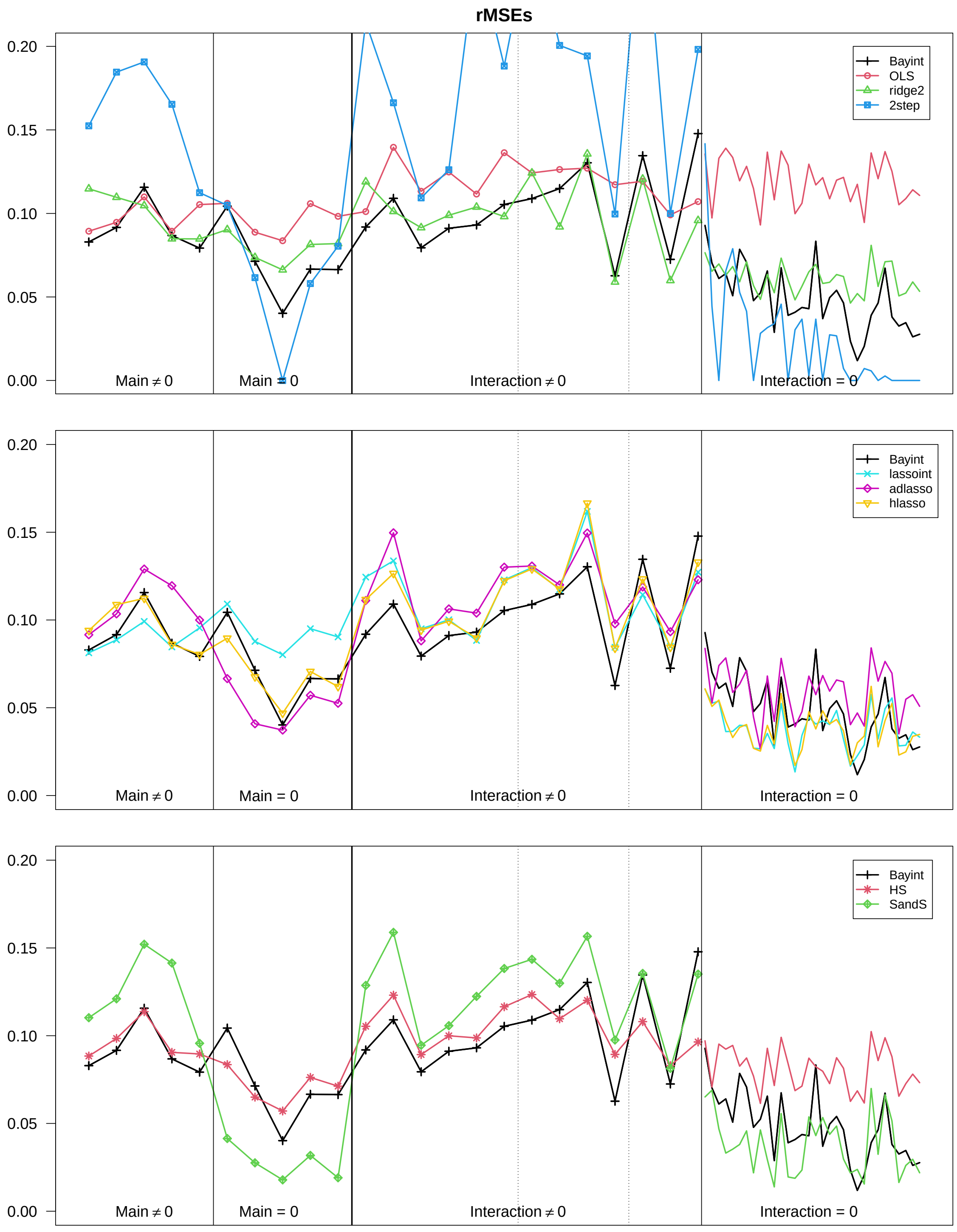

Figure 1: Simulation setting **1**. Root MSEs of the parameter estimates across $B = 50$ simulated data sets. Bold line demarcates main effects and interactions. Thin lines demarcates non-zero from zero effects. Dashed lines demarcate interactions corresponding to two (left), one (middle) and zero (right) non-zero main effects. Spacing for interactions with no effect adjusted to 1/4th for visual purposes

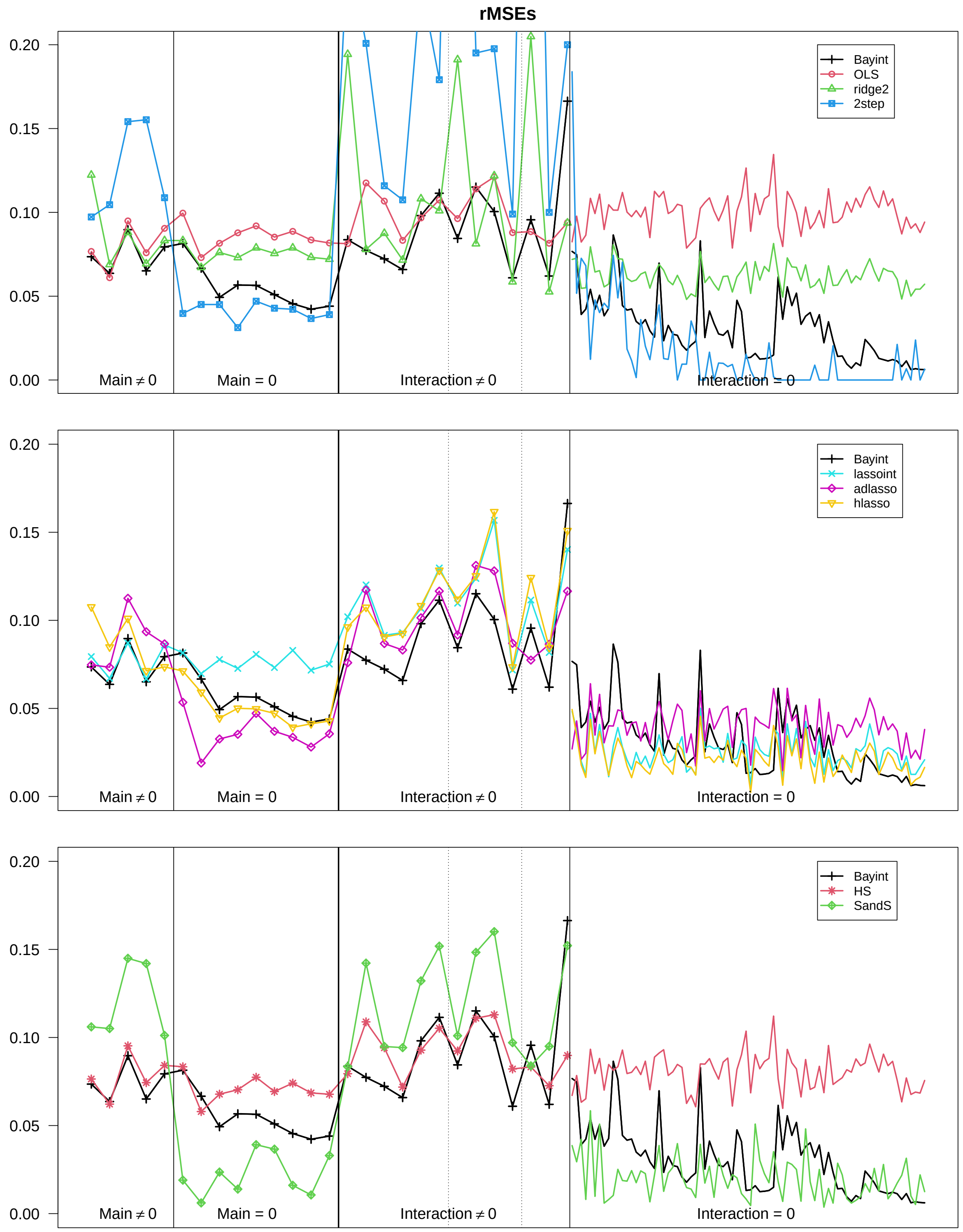

Figure 2: Simulation setting **2**. Root MSEs of the parameter estimates across $B = 50$ simulated data sets. Bold line demarcates main effects and interactions. Thin lines demarcates non-zero from zero effects. Dashed lines demarcate interactions corresponding to two (left), one (middle) and zero (right) non-zero true main effects. Spacing for interactions with no effect adjusted to 1/4th for visual purposes

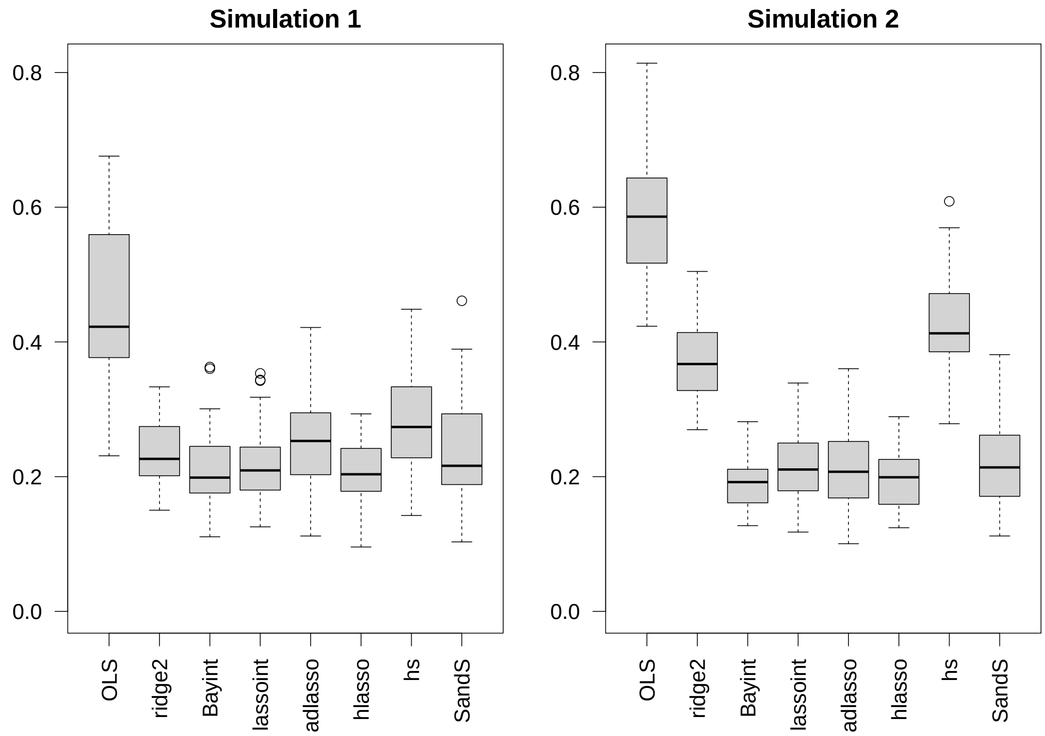

Figure 3: Mean squared errors of the predictions across $B = 50$ simulated data sets. Simulation settings 1 and 2.

3 Supplementary Figures and Tables for Real Data

Below we provide Supplementary Figures and Tables for the real (non-simulated) data. Further explanations are provided in the Main Document.

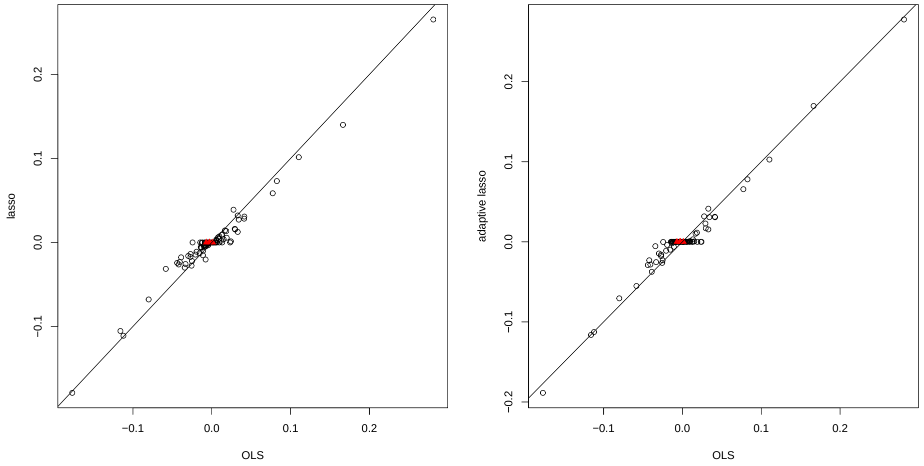

Figure 4: OLS estimates (x-axis) versus (adaptive) lasso estimates (y-axis) on the master set ($N = 21,570$). Red triangles: estimates for Noise main effects. Adaptive lasso uses OLS-based penalty weights to de-bias estimates of large coefficients. **Cholesterol** as outcome.

		Sensitivity				
FDR	Outcome	`Bayint`	`hlasso`	`adlasso`	`HS`	`SandS`
0.10	Chol	0.385	0.200	0.065	0.210	0.220
0.20	Chol	0.490	0.320	0.175	0.325	0.305
0.10	Sbp	0.100	0.140	0.015	0.070	0.075
0.20	Sbp	0.225	0.180	0.075	0.115	0.135

Table 2: Sensitivities for variable selection, interactions only, for fixed FDRs.

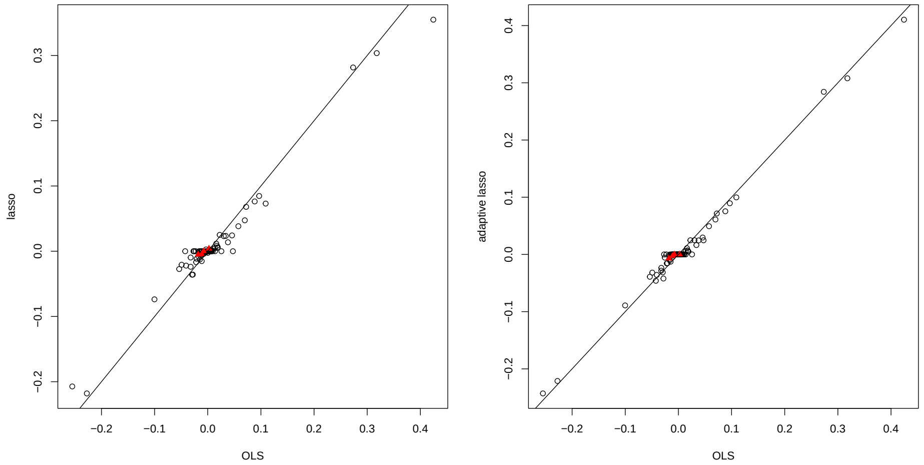

Figure 5: OLS estimates (x-axis) versus (adaptive) lasso estimates (y-axis) on the master set ($N = 21,570$). Red triangles: estimates for Noise main effects. Adaptive lasso uses OLS-based penalty weights to de-bias estimates of large coefficients. **SBP** as outcome.

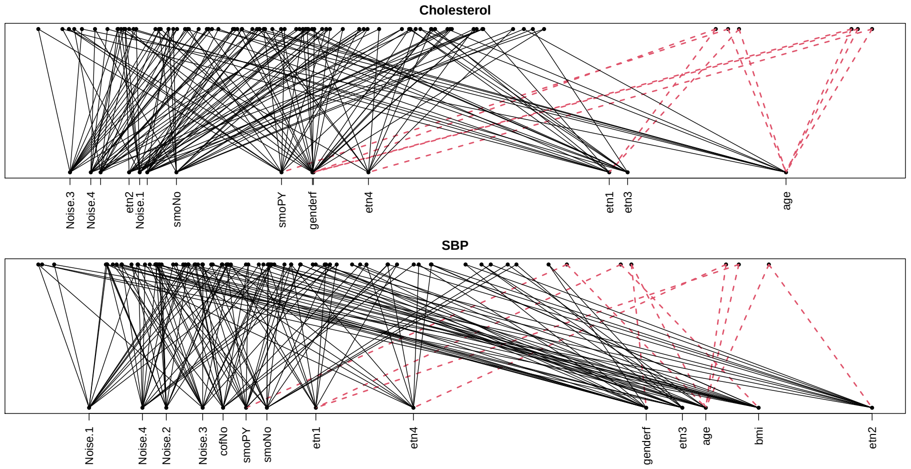

Figure 6: Connecting main effects to interactions for Cholesterol model (top) and SBP model (bottom). For each plot, lower line of dots shows square-root of absolute *true* coefficients (estimated from Master set) for main effects, top line for interactions (scaled to match the scale of the main effects). Interactions are connected with their corresponding main effects. Red dashed lines are used for the six largest interaction effects.

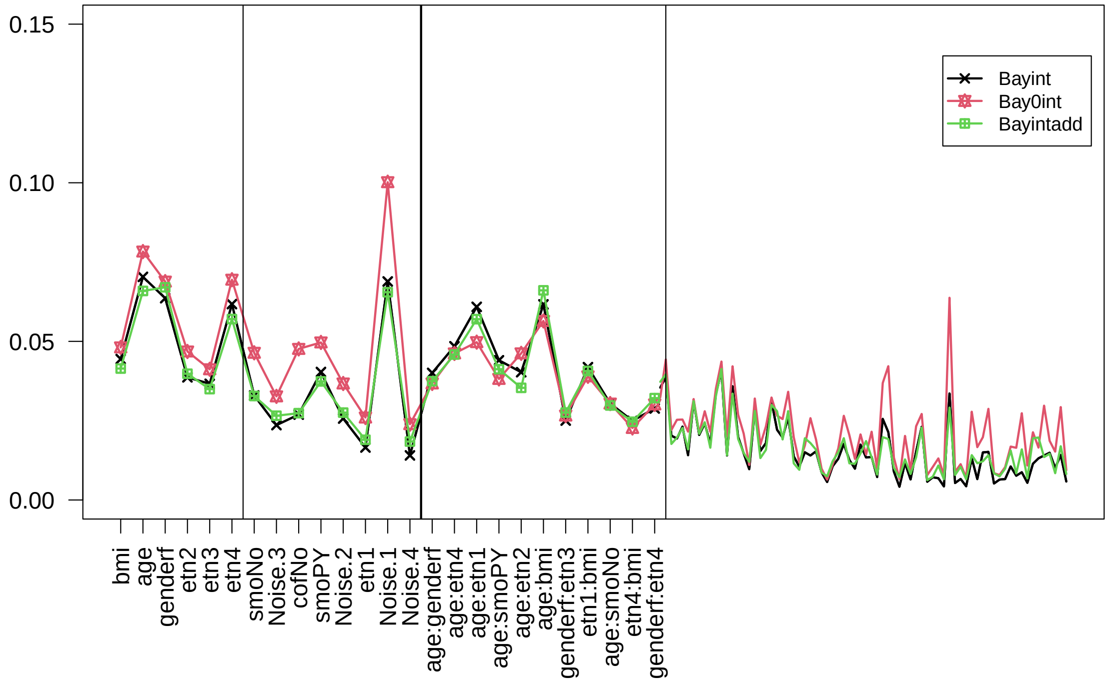

Figure 7: Comparing `Bayint` to `Bay0int` and `Bayintadd`. rMSEs for 14 main effects and 85 interactions (before and after bold vertical line), each ordered by significance in master set. Thin vertical line demarcates effects significant and non-significant effects in the master set (p<.01). **Cholesterol** as outcome. Spacing for weak interactions adjusted to 1/4th for visual purposes.

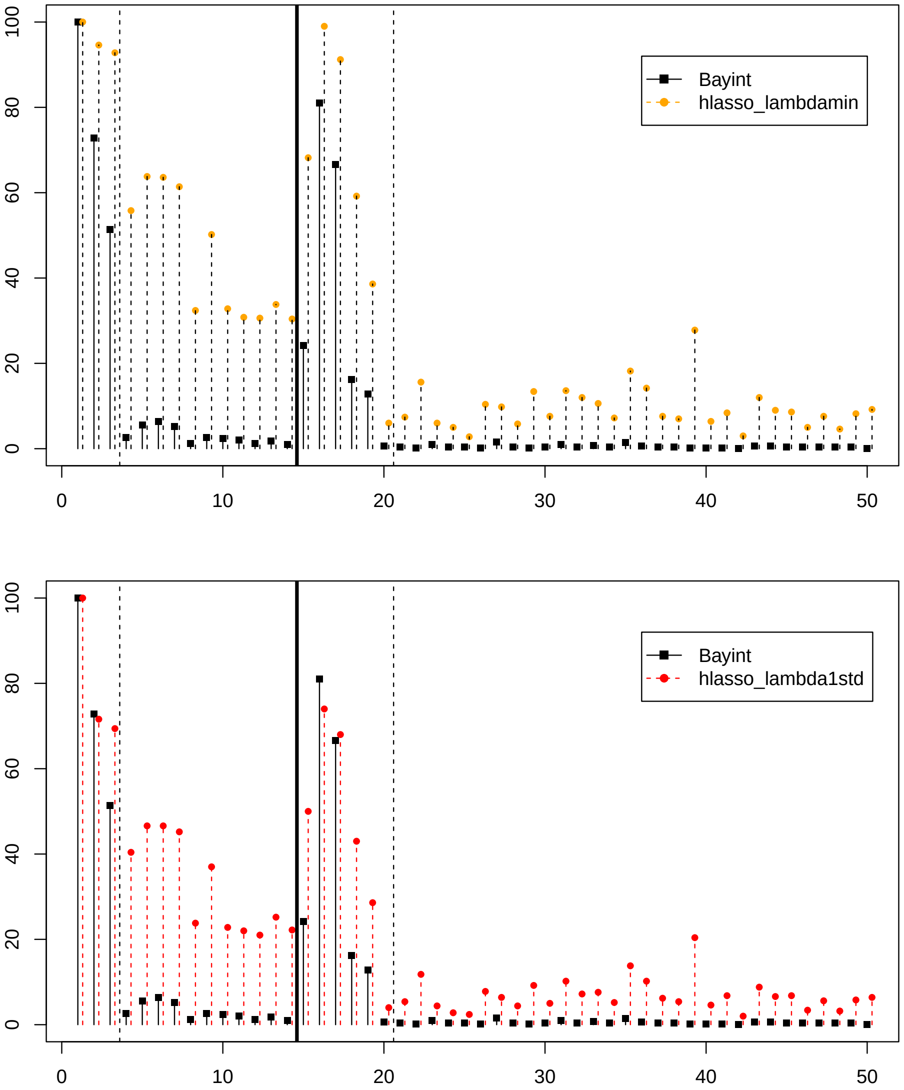

Figure 8: `Bayint` vs `hlasso`: Percentage detected effects (out of 500 random subsets of size $n = 1,000$), with covariates ordered according to absolute effect size in master set. Bold line demarcates main effects and interactions, dashed line separates effect sizes in master set larger and smaller than 0.075. Only first 50 are shown; remainder 49 behave similarly to $25, \ldots, 50$. **Cholesterol** as outcome.

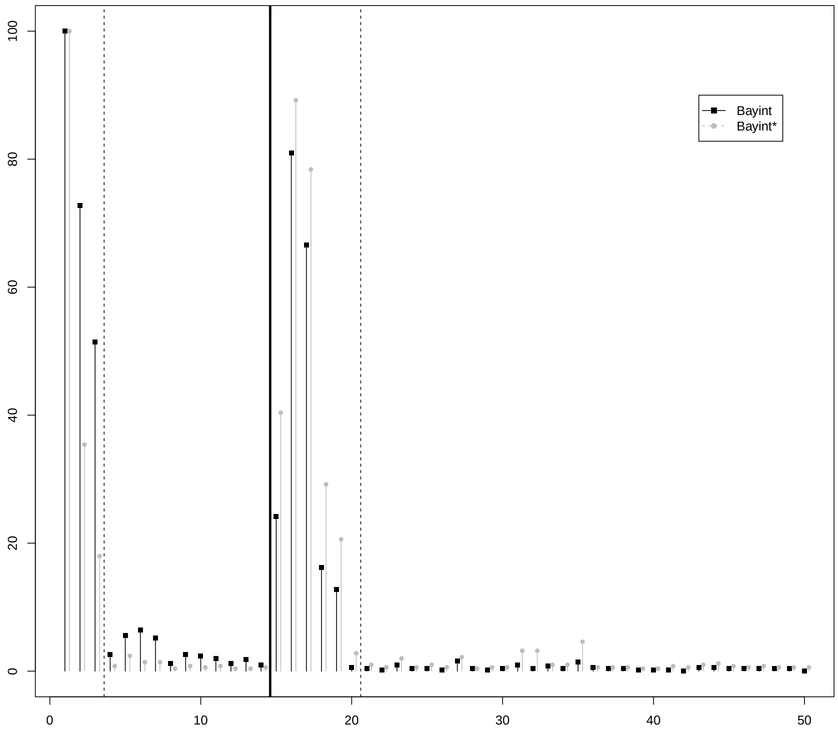

Figure 9: `Bayint` vs `Bayint*`: Percentage detected effects (out of 500 random subsets of size $n = 1,000$), with covariates ordered according to absolute effect size in master set. Bold line demarcates main effects and interactions, dashed line separates effect sizes in master set larger and smaller than 0.075. Only first 50 are shown; remainder 49 behave similarly to $25, \ldots, 50$. **Cholesterol** as outcome.

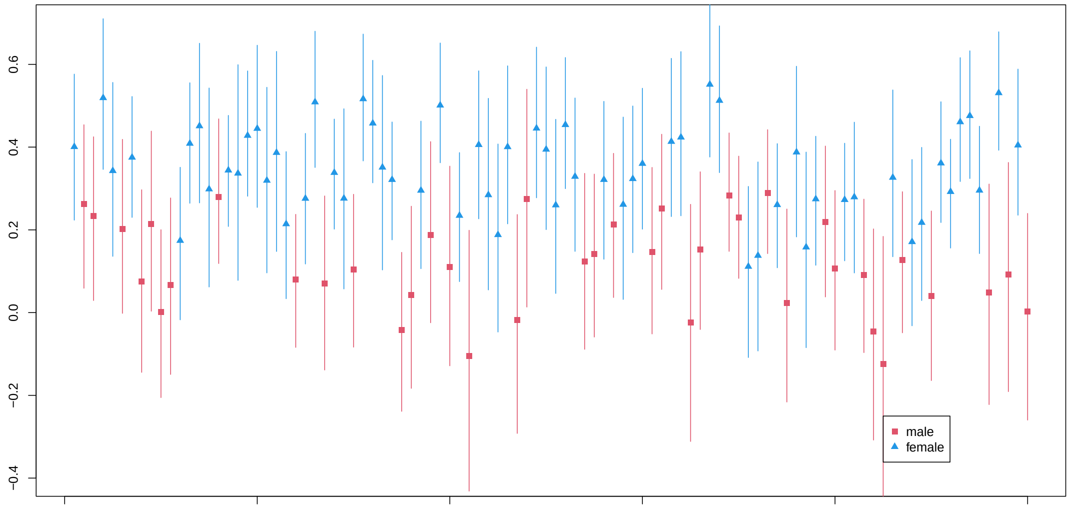

Figure 10: Effect of one unit age increase for males and females (100 random test samples, ordered by age) and its uncertainty, accounting for interactions of age with other covariates. `Bayint` model fitted on 1,000 training samples. **Cholesterol** (standardized) as outcome.

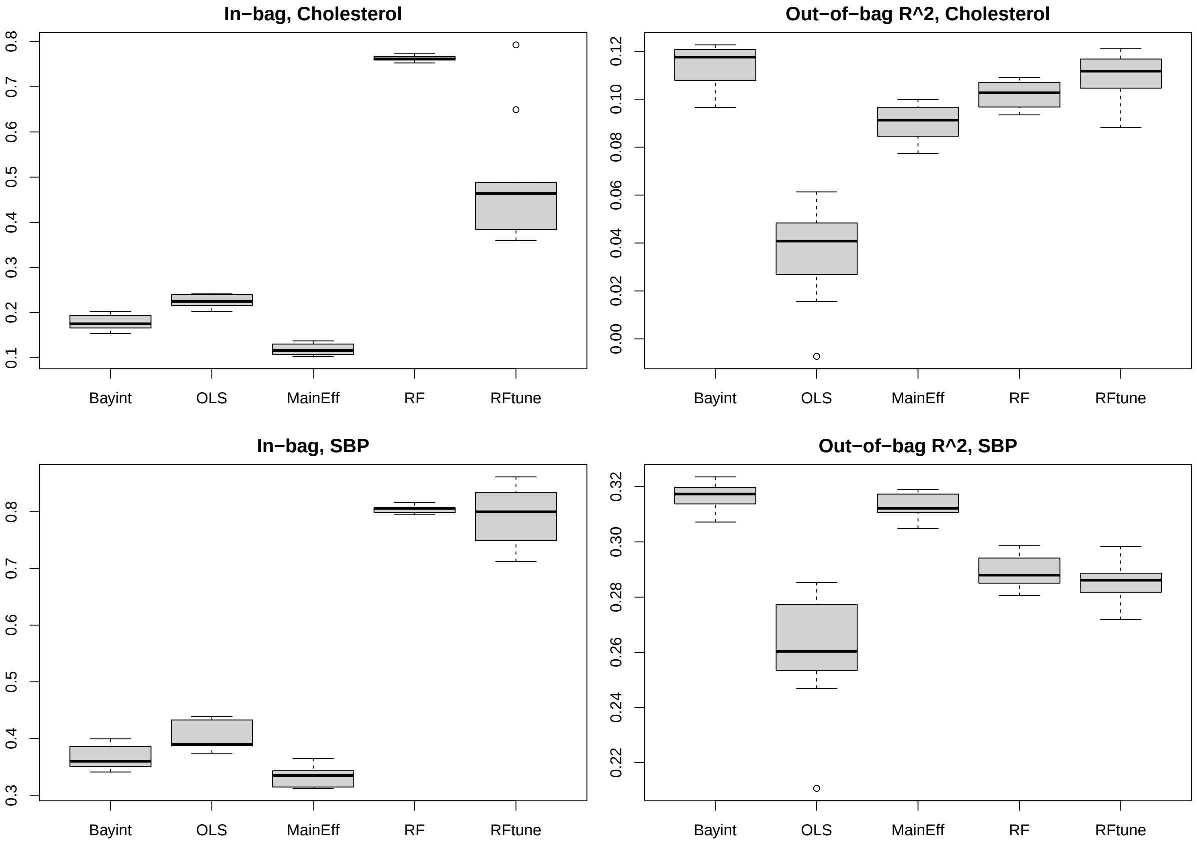

Figure 11: In-bag and out-of-bag R^2s for 25 training sets of size n=1,000 for cholesterol (top) and SBP (bottom) as outcome. Methods: `Bayint`: Bayesian linked shrinkage model; `MainEff`: OLS with main effects only; `RF` (`RFtune`): Random Forest with default (tuned) parameters.

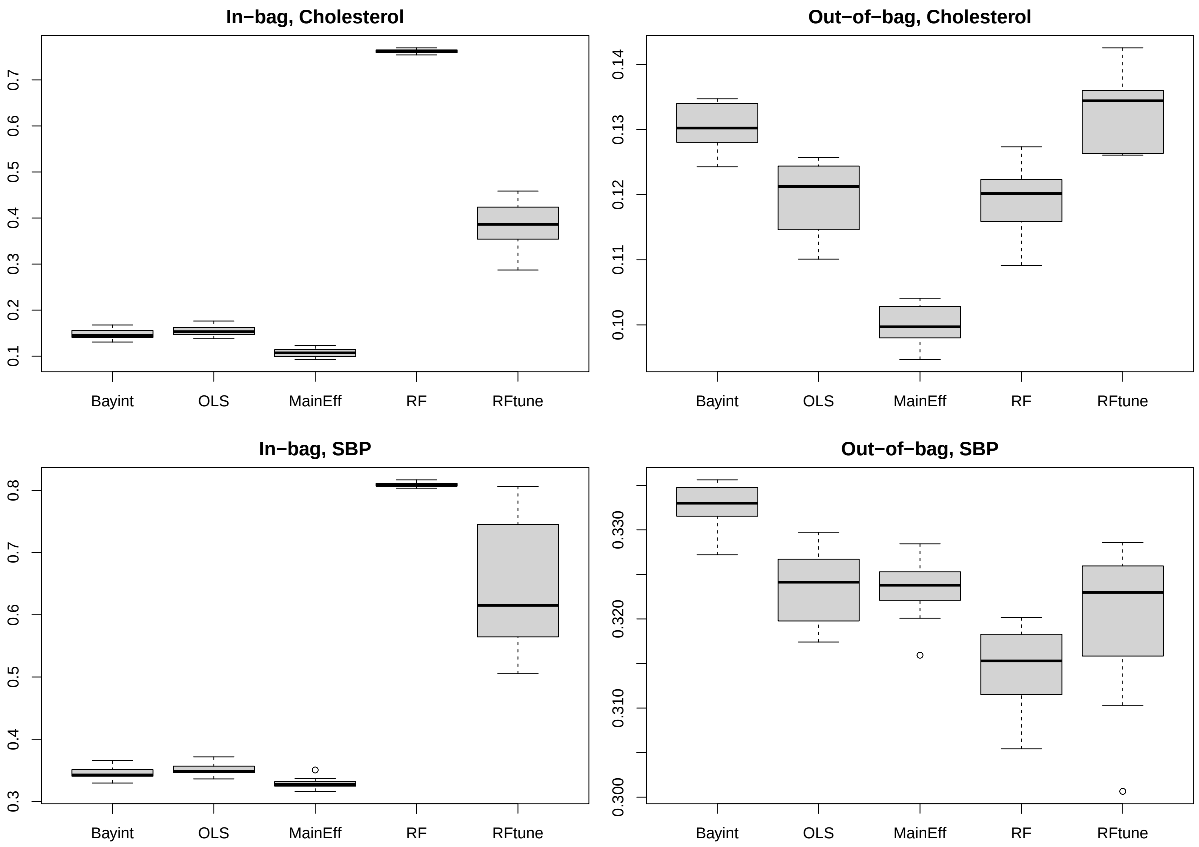

Figure 12: In-bag and out-of-bag R^2s for 10 training sets of size n=5,000 for cholesterol (top) and SBP (bottom) as outcome. Methods: `Bayint`: Bayesian linked shrinkage model; `MainEff`: OLS with main effects only; `RF` (`RFtune`): Random Forest with default (tuned) parameters.

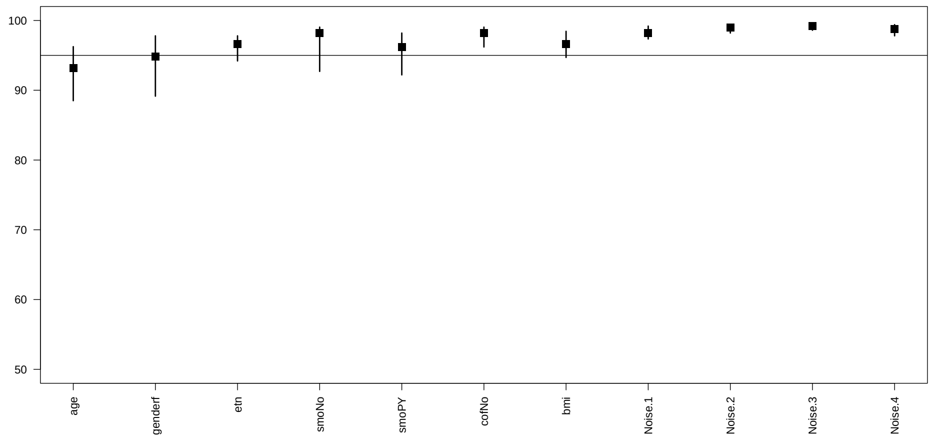

Figure 13: Coverages of 95% credible intervals for Shapley values. Estimated Shapley's and intervals are obtained from 500 random subsets of size $n = 1,000$. True Shapley values are based on parameter estimates from the master set. All Shapleys's are computed for 200 random test individuals. For the test set median, first and third quartile coverage are shown. **Cholesterol** as outcome.

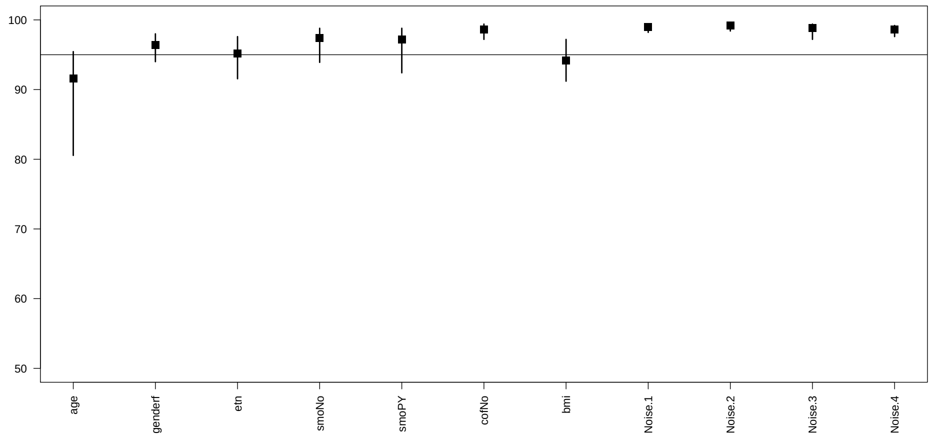

Figure 14: Coverages of 95% credible intervals for Shapley values. Estimated Shapley's and intervals are obtained from 500 random subsets of size $n = 1,000$. True Shapley values are based on parameter estimates from the master set. All Shapleys's are computed for 200 random test individuals. For the test set median, first and third quartile coverage are shown. **Sbp** as outcome.

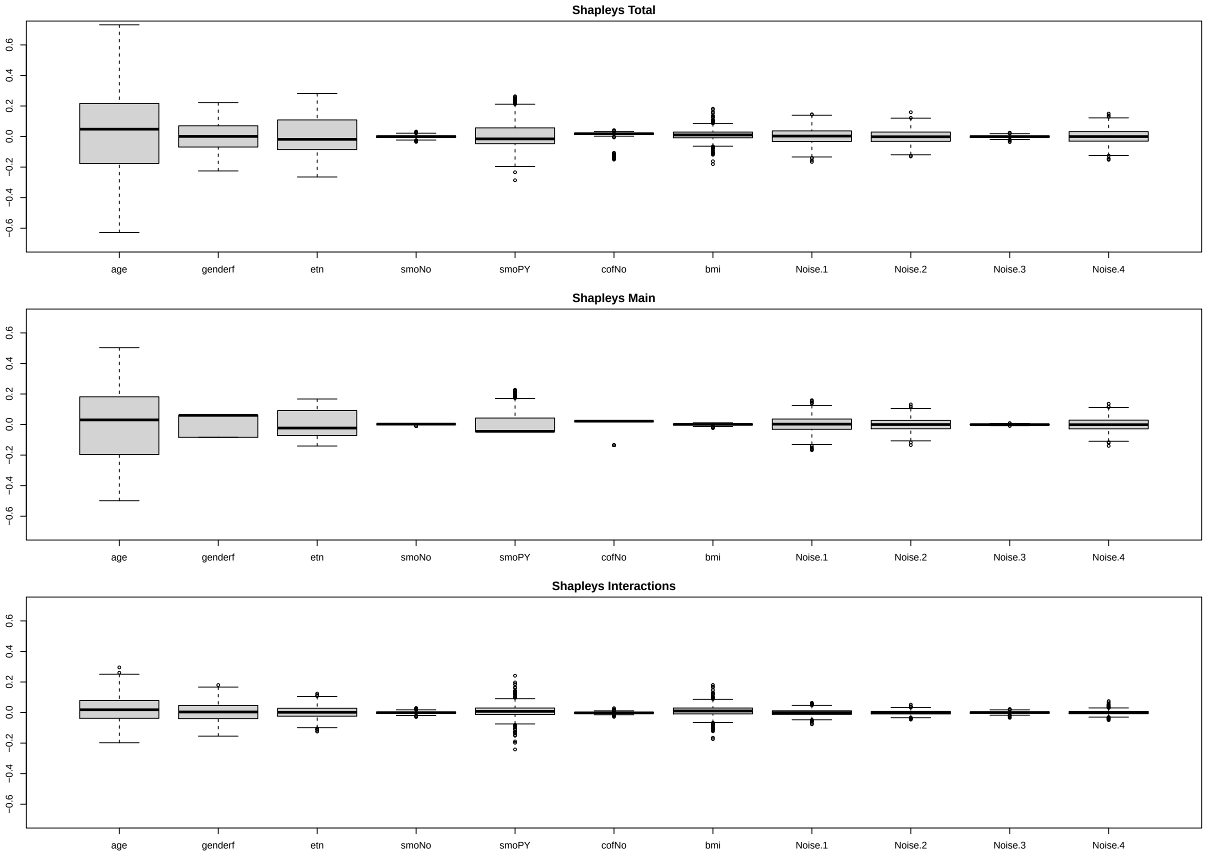

Figure 15: Distribution of Shapley values of all covariates over 1,000 random test individuals. **Cholesterol** (standardized) as outcome.

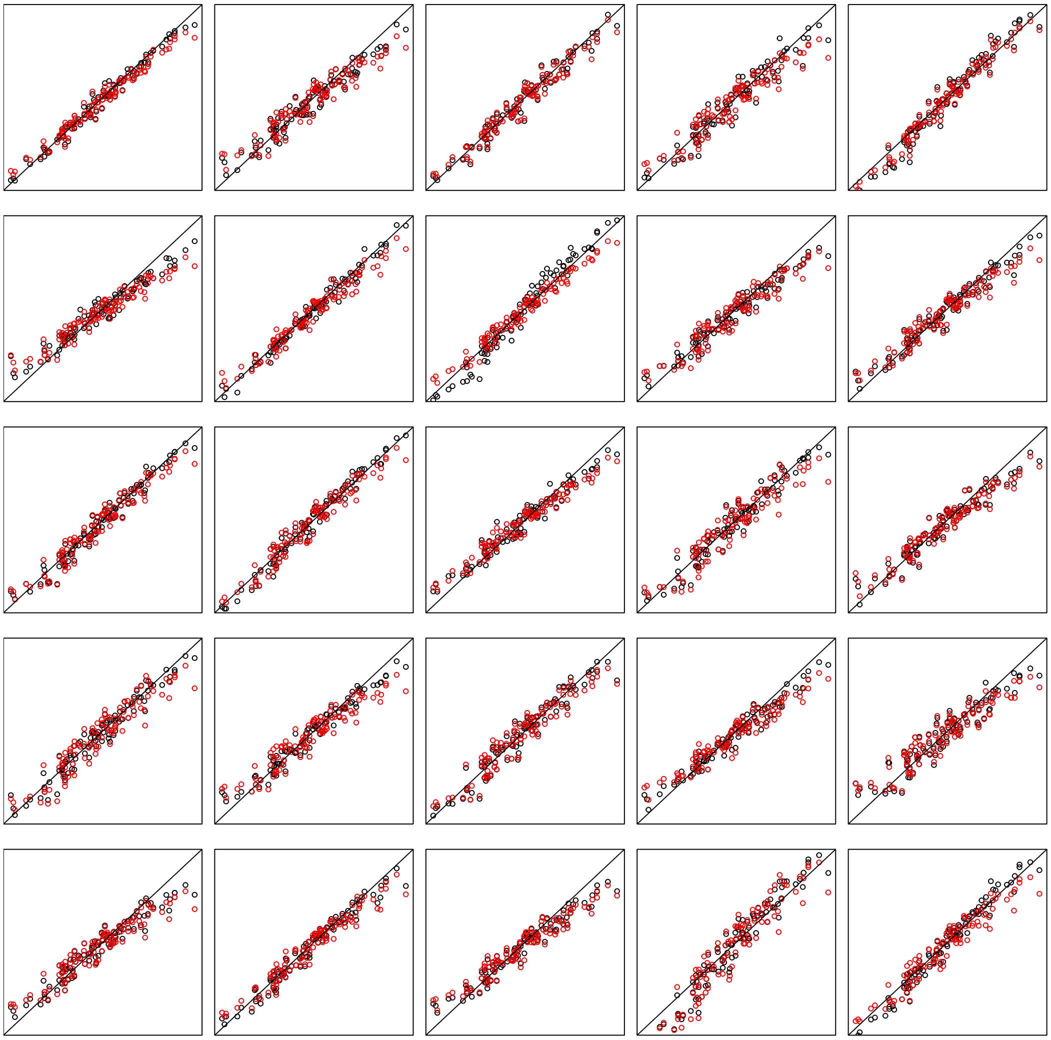

Figure 16: Shapley values for 'age' over 1,000 random test individuals (dots) for 25 training sets (displays). X-axis: true Shapley values; Y-axis: estimated ones by `Bayint` (black) and `hlasso` (red). **Cholesterol** (standardized) as outcome.

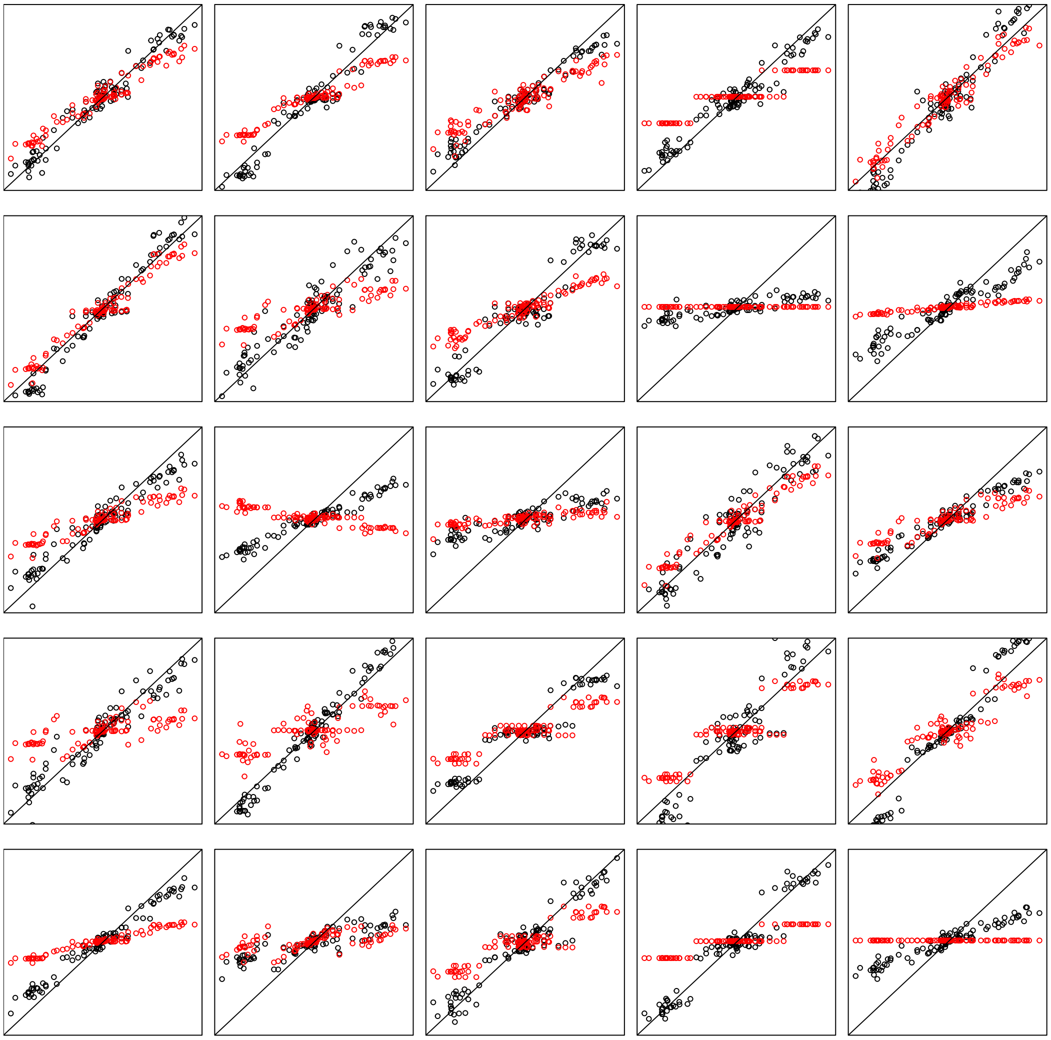

Figure 17: Shapley values for 'etn1' over 1,000 random test individuals (dots) for 25 training sets (displays). X-axis: true Shapley values; Y-axis: estimated ones by `Bayint` (black) and `hlasso` (red). **Cholesterol** (standardized) as outcome.

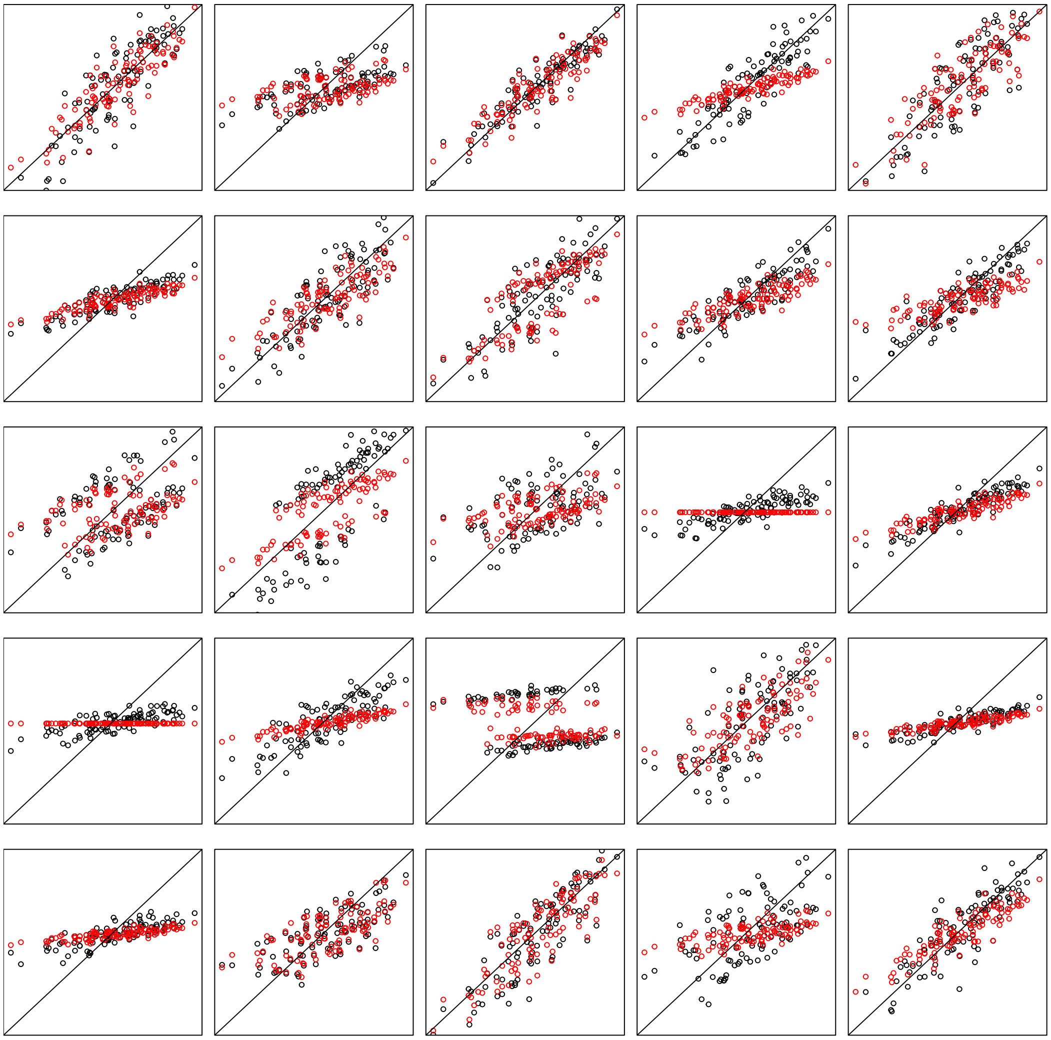

Figure 18: Shapley values for ‘gender’ over 1,000 random test individuals (dots) for 25 training sets (displays). X-axis: true Shapley values; Y-axis: estimated ones by `Bayint` (black) and `hlasso` (red). **Cholesterol** (standardized) as outcome.

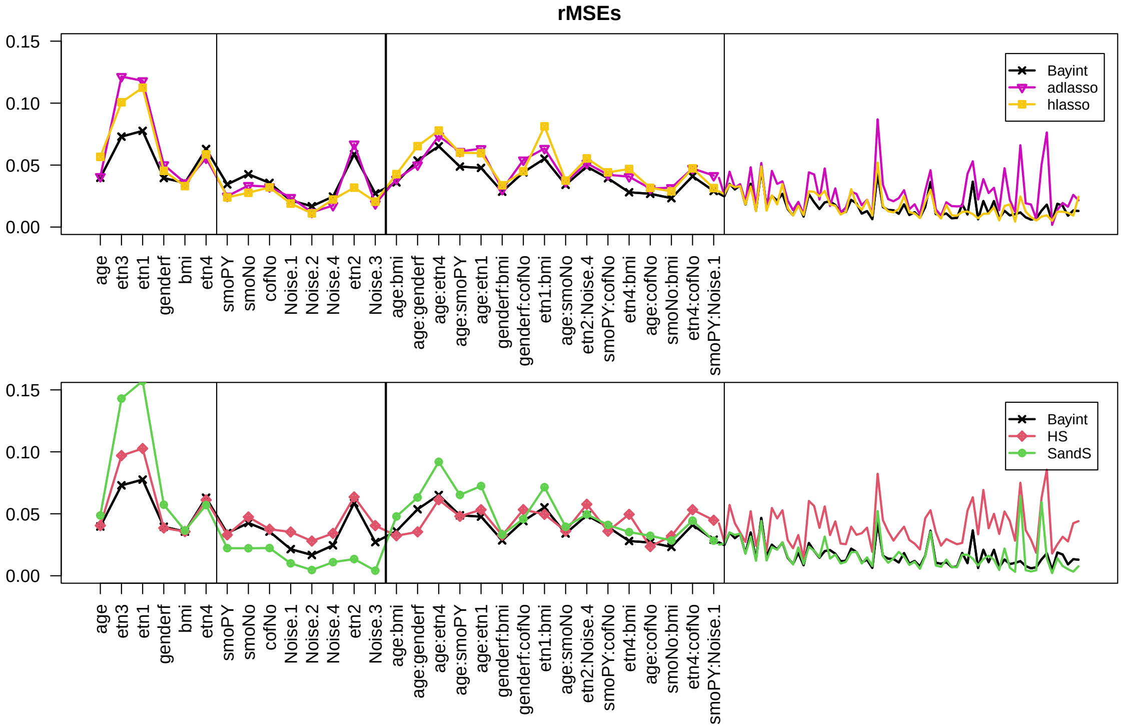

Figure 19: rMSEs computed from *synthetic* data set for 14 main effects and 85 interactions (before and after bold vertical line), each ordered by significance in synthetic master set. Thin vertical line demarcates effects significant and non-significant effects in the synthetic master set ($p<.01$). **Cholesterol** as outcome. Spacing for weak interactions adjusted to 1/4th for visual purposes. Same methods evaluated as for the real data set.

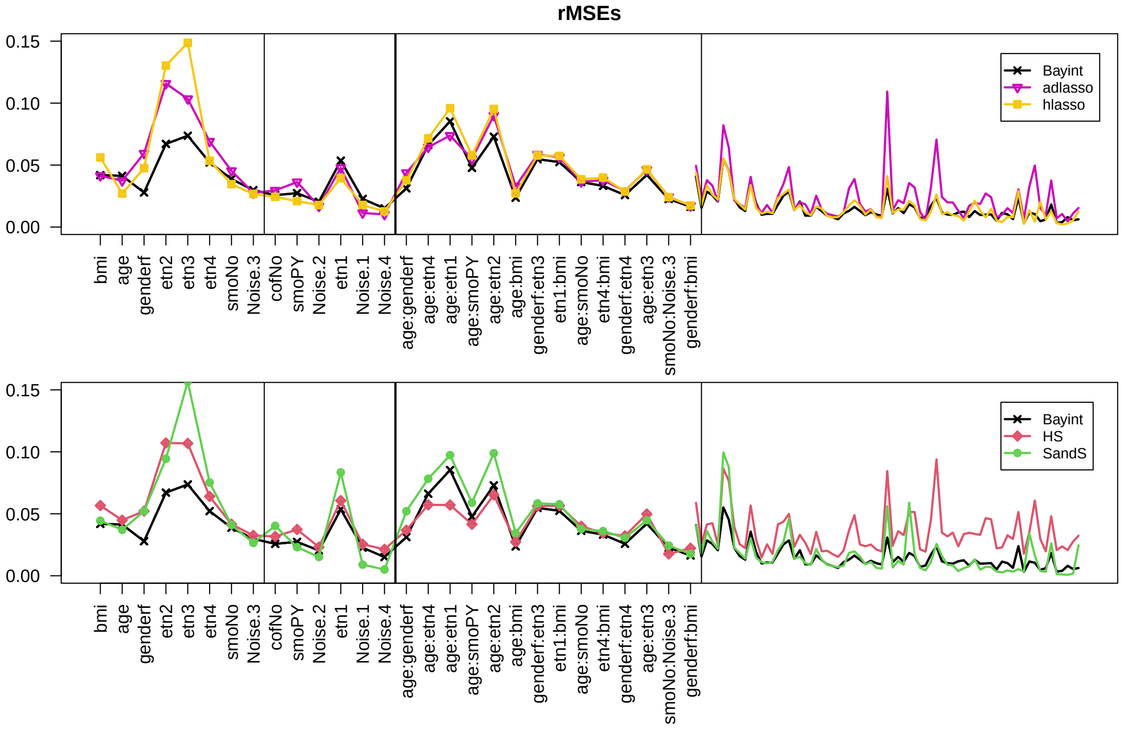

Figure 20: rMSEs computed from *synthetic* data set for 14 main effects and 85 interactions (before and after bold vertical line), each ordered by significance in synthetic master set. Thin vertical line demarcates effects significant and non-significant effects in the synthetic master set (p<.01). **SBP** as outcome. Spacing for weak interactions adjusted to 1/4th for visual purposes. Same methods evaluated as for the real data set.
